# An economical and highly adaptable optogenetics system for individual and population-level manipulation of *Caenorhabditis elegans*

**DOI:** 10.1186/s12915-021-01085-2

**Published:** 2021-08-24

**Authors:** M. Koopman, L. Janssen, E. A. A. Nollen

**Affiliations:** European Research Institute for the Biology of Ageing, University of Groningen, University Medical Center Groningen, Groningen, The Netherlands

**Keywords:** OptoArm, Optogenetics, *Caenorhabditis elegans*, Neuronal ageing, Rhodopsin, Worm trackers

## Abstract

**Background:**

Optogenetics allows the experimental manipulation of excitable cells by a light stimulus without the need for technically challenging and invasive procedures. The high degree of spatial, temporal, and intensity control that can be achieved with a light stimulus, combined with cell type-specific expression of light-sensitive ion channels, enables highly specific and precise stimulation of excitable cells. Optogenetic tools have therefore revolutionized the study of neuronal circuits in a number of models, including *Caenorhabditis elegans*. Despite the existence of several optogenetic systems that allow spatial and temporal photoactivation of light-sensitive actuators in *C. elegans*, their high costs and low flexibility have limited wide access to optogenetics. Here, we developed an inexpensive, easy-to-build, modular, and adjustable optogenetics device for use on different microscopes and worm trackers, which we called the OptoArm.

**Results:**

The OptoArm allows for single- and multiple-worm illumination and is adaptable in terms of light intensity, lighting profiles, and light color. We demonstrate OptoArm’s power in a population-based multi-parameter study on the contributions of motor circuit cells to age-related motility decline. We found that individual components of the neuromuscular system display different rates of age-dependent deterioration. The functional decline of cholinergic neurons mirrors motor decline, while GABAergic neurons and muscle cells are relatively age-resilient, suggesting that rate-limiting cells exist and determine neuronal circuit ageing.

**Conclusion:**

We have assembled an economical, reliable, and highly adaptable optogenetics system which can be deployed to address diverse biological questions. We provide a detailed description of the construction as well as technical and biological validation of our set-up. Importantly, use of the OptoArm is not limited to *C. elegans* and may benefit studies in multiple model organisms, making optogenetics more accessible to the broader research community.

**Supplementary Information:**

The online version contains supplementary material available at 10.1186/s12915-021-01085-2.

## Background

Neurotransmission is defined as the process by which neurons transfer information via chemical signals at synaptic contacts (e.g., synapses) with target cells. Those target cells can be other neurons, but also non-neuronal cell types (e.g., muscle cells). *Caenorhabditis elegans* has proven to be an important model organism to study fundamental neurobiology, including neurotransmission at chemical synapses [1–18]. As a matter of fact, important molecular players involved in synaptic transmission, synaptic vesicle docking, priming, fusion, and recycling have actually been discovered in *C. elegans* and are highly conserved in mammalian systems [12, 19–24]. Initially, pharmacological assays were predominantly used to analyze synaptic transmission and its associated molecular substrates in *C. elegans* and to determine whether an observed neurotransmission defect was pre- or postsynaptic of nature [3, 9]. Subsequently, the development of electrophysiology resulted in new techniques that have allowed researchers to study the electrical events that occur at synapses more directly [11–17, 25–30]. These methods provide a quantifiable readout for the underlying synaptic activity and a way to investigate synaptic properties. Nevertheless, standard electrophysiology in *C. elegans* is technically challenging and cannot be performed in intact worms, making it impossible to directly correlate synaptic activity with behavioral readouts [31]. The development of optogenetics, however, addressed this issue and created the possibility to directly manipulate synaptic activity and simultaneously look at behavioral changes [31–36].

**Fig. 1:**
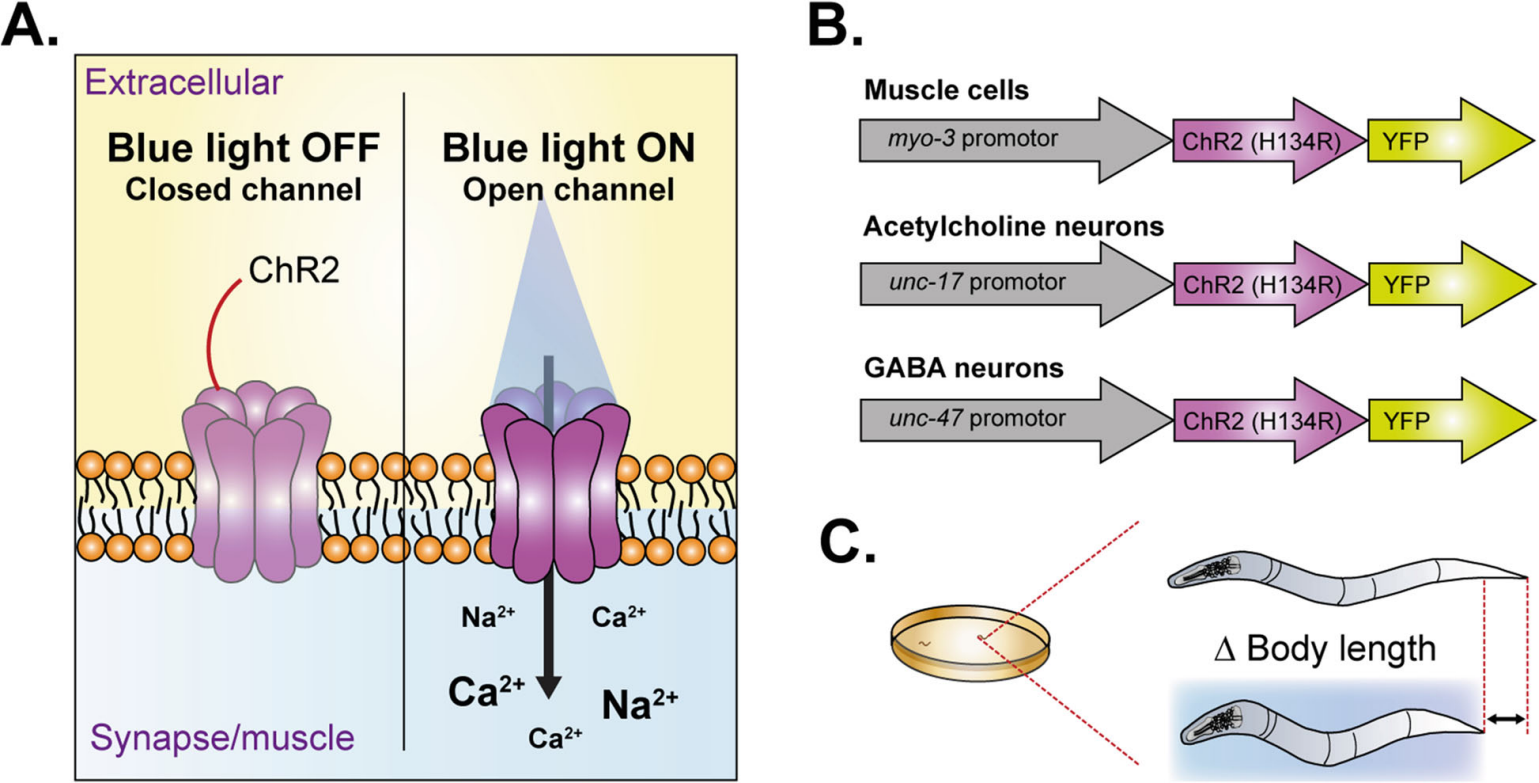
Channelrhodopsin-2 is a light-sensitive cation channel. **A** Channelrhodopsin-2 (ChR2) is rapidly opened by stimulation with blue (440–460 nm) light in an essentially nondesensitizing manner. ChR2 is permeable by multiple cations like Na^+^ and Ca^2+^ and enables strong and rapid membrane depolarization of the cell it is expressed in. **B** Multiple ChR2-expressing *C. elegans* strains exist, including but not limited to expressing in muscle cells (*myo-3*), cholinergic neurons (*unc-17*), and GABAergic neurons (*unc-47*). **C** The change in body length is often used as a readout of ChR2 activation (see B)

Optogenetics is based on the genetic expression of a light-sensitive actuator that can actively influence biochemical reactions or neuronal activity in response to a noninvasive light stimulus [31, 37]. The most common used “actuators” are the rhodopsins, which have been discovered in algae, and are now widely used in different cells and organisms to depolarize or hyperpolarize cells upon light stimulation [32, 37–43]. The transparent body of *C. elegans*, its amenability to genetic manipulation and its invariant nervous system, make the worm ideal for optogenetic manipulation [31, 44–46]. In fact, *C. elegans* was the first multicellular organism to have its behavior manipulated in vivo by the photoactivation of channelrhodopsin-2 (ChR2) in muscle cells [32]. ChR2 is a light-sensitive cation channel that can undergo a light-induced conformational change, thereby allowing H^+^, Na^+^, K^+^, and Ca^2+^ ions to passively diffuse down their concentration gradients (Fig. 1A) [32, 38, 47]. In excitable cells, this results in rapid depolarization of the plasma membrane and the subsequent initiation of downstream events, like fibril contraction in muscle cells and synaptic vesicle release in neuronal cells [32–34, 36, 37, 48–50]. The specificity of the response is acquired by expressing optogenetic proteins under specific promoters (Fig. 1B) in the worm. For instance, the expression of ChR2 in motor neurons (*unc-47* and *unc-17* promotors) or muscles (*myo-3* promotor) elicits either a flaccid or spastic paralysis upon complete illumination of the worms [31, 33, 37]. This obvious and robust response results in a change in body length that can be used as a clear readout (Fig. 1C) [33, 37]. Multiple other strains exist, in which for example specific interneurons or sensory neurons are under the control of ChR2 [32, 51–62]. Functional ChR2 requires the chromophore all-trans retinal (ATR), which is not endogenously produced by *C. elegans* but can be supplied via exogenous feeding [32].

Over the years, multiple systems have been developed to illuminate *C. elegans* in a temporal and/or spatial manner for optogenetic experiments. Most of those systems are custom built and include fluorescence microscopes, or systems with lasers and shutters [57, 58, 63–68]. These systems require costly equipment and are mostly designed for single-animal illumination. While the costs might limit the general use of these systems for a wider audience, they do, however, provide clear benefits for fundamental research requiring single-cell stimulation. For example, some of these systems provide ways of following single worms in space and time with chromatic precision, thereby ensuring exact illumination of a specified anatomical position [57, 58, 68, 69]. This targeted illumination makes it possible to optogenetically excite single neurons for which specific promotors are not known [69]. Moreover, in these expensive systems, one can adjust the light intensity relatively easily, making the systems flexible and adaptable for different types (e.g., short- or long-term) of experiments.

Several researchers have tried to make optogenetical experiments more accessible to a wide audience by successfully developing inexpensive (< $1500) optogenetic systems [70–74]. Some of these low-cost systems even allow multiple worms to be illuminated at the same time [71, 74]. When considering the current developments of automated worm trackers [57, 58, 75–83], the ability to illuminate multiple worms at the same time during tracking offers a clear benefit for studying both population and single-worm characteristics. However, there are also clear limitations of these low-cost systems. In fact, most of the systems require manual adaptations (e.g., aluminum foil, closed boxes) to ensure a light intensity that is high enough (e.g., at least 1.0 mW/mm^2^) for optogenetic purposes in *C. elegans* [72, 74]. These adaptations make the simultaneous tracking of illuminated worms challenging and highly reduce the adaptability of the system to other experimental set-ups. Moreover, within low-cost systems, the light source often has to be placed in very close proximity (< 2 cm) to the worms, which makes such systems unsuitable for long-term stimulation due to potential heat generation and phototoxicity [71, 72, 74]. Some systems, such as the OptoGenBox [84], are exempt from these issues, but they are also more expensive.

Clearly, there is no shortage of tools and techniques to perform optogenetic experiments with *C. elegans*. Ideally, one would simply look at all the advantages and limitations of each system and select for the platform that fits best to the biological questions to be answered. Practically, however, there are often different types of biological questions to be answered or various approaches required to tackle a specific hypothesis. We argue that the use of optogenetics should not be restricted by limited capabilities of a single system or available (financial) resources. From that perspective, there remains a challenge in developing low-cost optogenetic devices with high flexibility and adaptability. Here, we describe in detail how we developed a sub-$100 optogenetic device, the OptoArm. This system provides the user with an easy-to-build, highly adaptable, flexible, and reliable optogenetic system. The arm allows integration in different experimental set-ups and tackles both single- and multiple-worm illumination, making combinatorial approaches and population-based studies possible. As proof-of-principle, we demonstrate the use of the OptoArm by determining the functional contribution of motor circuit cells to the age-related decline in motility. Finally, by offering both the option to build a manual and a fully automated system, researchers are able to pick the set-up that fits their budget and requirements best. The biologically and technically validated OptoArm makes optogenetics experiments accessible for researchers both within and outside the laboratory, including teaching institutions.

## Results

### Efficient thermal management is key in constructing a compact, inexpensive set-up

#### Construction

**Fig. 2:**
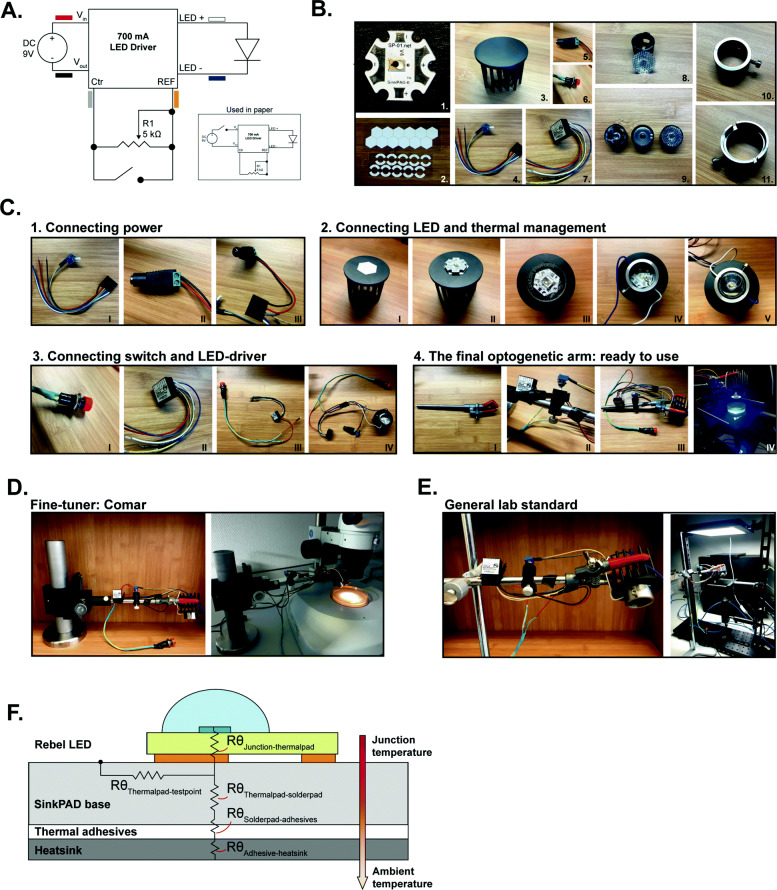
Building the OptoArm only requires basic knowledge of electronics and thermal management. **A** The optimal electronic circuitry of the OptoArm. Inset: the variant of the circuitry used in this paper to test and validate the system. The color codes refer to the wires of the LED driver that is used in this paper. **B** The essential components required to build the electronic circuitry of the OptoArm: 1. Luxeon Rebel LED, 2. Thermal adhesives, 3. Heatsink, 4. Wire harness with potentiometer, 5. DC plug, 6. ON/OFF button, 7. LED driver, 8. lens with case, 9. Several lenses, 10. and 11. Lens holder. **C** The steps required to construct the OptoArm, detailed instructions per picture can be found in Table 3. *STEP 1:* connecting the solderless DC plug to the connecting wire harness. *STEP 2*: mounting the LED on a heatsink and soldering the connecting wire harness to the LED cathode and anode. *STEP 3*: Connecting the ON/OF switch to the LED driver and connecting the driver to the wire harness. Note, the pictures show a serial set-up, and not the recommend parallel wiring (see 2A, main). *STEP 4*: mounting the electronic circuitry to a standard flask clamp to finish the OptoArm. By placing the OptoArm in either a **D** fine-tuner or a **E** general lab standard, the system can be used for different applications. **F** The different thermal resistances (*Rθ*) in the heat flow between the LED junction, temperature test point, and bottom of the LED assembly

To build a simple and inexpensive optogenetics device that can be adjusted to laboratory-specific parameters, specific applications and requirements, we investigated the use of low-cost, high-intensity LEDs with interchangeable optics. The ease of the construction and the low costs of the system ensures accessibility for both researchers and students. We opted for a straightforward electronic circuitry (Fig. 2A) using a 700-mA LED driver to ensure the feeding of fixed electric current to a 1.03-W LED with a wavelength ranging between 440 and 460 nm (optimal: 448 nm) (see Table 1 for a basic introduction in electronics). The placement of the ON/OFF switch between the CTRL and REF pins, in parallel with the potentiometer (Fig. 2A, main panel), provides the best management of inrush power and spikes to the LED. Therefore, this is the recommended configuration to ensure an optimal lifetime for the LED. For prototyping purposes, however, we used a circuit with the switch serially connected to the LED driver and the DC plug (Fig. 2A, insert). As depicted in Fig. 2B, in addition to the high-intensity LED and the LED driver, only a small set of components is required (see Table 2 and “Methods”): a potentiometer to adjust intensity, an ON/OFF switch, a lens holder (home-made) with interchangeable lenses, a heatsink, and thermal adhesives to connect the components (see “Thermal management” section) and a 9V_DC_ adapter to power the system. The different steps required to build the optogenetic arm can be found in Fig. 2C and Table 3. Eventually, we installed the complete circuitry on a standard flask clip clamp, as this could be used in combination with different stands for different set-ups (Fig. 2D, E, Table 2). Note that the combination of the wavelength and high intensity makes the LED dangerous for the human eye. We should try to avoid looking directly into the LED, use protective sheets, or wear protective glasses.

**Table 1:** A basic introduction of circuit components

While it is beyond the scope of this paper to provide an introduction into electronics, we want to provide some basic background information about the electronic components and their function in the circuit. However, more detailed information is readily available on the internet or in any electronics handbook.	
• **LED** (light-emitting diode):	This component is a semiconductor that converts the energy of exited electrons into photons to produce light. One important characteristic of LEDs is their polarity, i.e., they have a positive and negative side. In order for the LED to function, it is paramount to connect its anode (+) to the positive side of the power supply and the cathode (−) to the negative side, so the current will flow in the right direction. Other key characteristics of LEDs are their tendency to produce heat and their sensitivity to heat. While you may have heard LEDs produce no heat and can be cool to the touch, substantial heat gets produced inside the device and this can change the electrical properties of the semiconductor. In the short term, this can decrease the output of the LED, but heat will also significantly reduce the LED’s lifetime or damage the LED permanently. In fact, the change in characteristics upon heating can give rise to “Thermal Runaway,” where the rise in temperature causes the resistance to lower and the LED to draw more current, leading to a further increase in heat production. This vicious circle will eventually cause the LED burnout. To prevent this from happening, it is important to power the LED with constant current.
• **LED driver**:	This component transforms the power from the power supply and delivers a constant current to the LED to prevent “Thermal Runaway.” The DC model used in our set-up has 6 leads. A red/black pair and a white/blue pair are attached to the positive and negative leads of the power supply and LED, respectively. In addition, it has an internal reference lead putting out 5 V DC (yellow) and a control input lead (gray). These last two leads allow external control over the current output and, by extension, the brightness of the LED.
• **Potentiometer**:	This is a passive electronic component that has a variable resistance. By placing a potentiometer between the internal reference and the control input, it is possible to regulate how much of the 5 V from the reference gets delivered to the control input. There is an inverse relationship between the voltage supplied to the input control and the current output of the driver, i.e., 5 V to the input will reduce output current to zero and 0 V will yield the maximum output.
• **Microcontroller**:	This is essentially a small computer. It can be configured to receive input signals, process them and return output signals. In our automated set-up, an Arduino Uno microcontroller is used to replace the potentiometer. This allows a more precise regulation of the LED intensity and timing of light pulses. In this set-up, the 5 V internal reference is used to power the Arduino and one of Arduino’s pulse width modulation (PWM) pins is used to supply voltage to the input control. Arduino pins can generally only switch between an off (0 V) and on (5 V) state. However, PWM pins can cycle rapidly between these on and off states to generate an output voltage between 0 and 5 V that is perceived as continuous, simulating the effect of the potentiometer.
• **Capacitor**:	This is a passive electronic component that stores electrical energy. Applying a voltage over a capacitor charges an electric field within the capacitor. In our automated set-up, a bypass capacitor is introduced, connecting the internal reference to the ground. The DC signal provided by the LED driver may not be “pure” DC and can display some high-frequency noise fluctuations, which can cause erratic behavior of the microcontroller. The capacitor will counteract these fluctuations by charging or discharging accordingly.

**Table 2:** Different variants of the OptoArm, their requirements and costs

	Minimal requirements OptoArm^**a**^ (Fig. **2**)	Minimal requirements OptoArm with low-cost stand^**a,b**^ (Fig. **2**)	Automated OptoArm with low-cost stand^**a**^ (Fig. **10**)	Additional improvements or available changes^**a, d**^
**Electronics**	• Royal-Blue (448 nm) Rebel LED • 700 mA externally dimmable BuckPuck DC driver • Connecting wire with adjustable potentiometer of 5 kΩ • Latching pressure switch (ON/OFF switch) • DC contra plug • 9V_DC_ – Adapter	**All the previously mentioned components**	**All the previously mentioned components** **Except:** • Connecting wire with adjustable potentiometer of 5 kΩ **Plus:** • Arduino Uno (or clone) • Capacitor (0.1 μf) • Adafruit LCD shield	**Yellow LED for NpHR/Halo/Mac**^**e**^**:** e.g., Lime (567 nm) Rebel LED on a SinkPAD-II 20 mm Star Base - 368 lm @ 700 mA COST: $11 **Tri-star LED for higher intensity**^**e**^**:** Royal-Blue (448 nm) Rebel LED on a SinkPAD-II 20 mm Tri-Star Base - 3090 mW @ 700 mA. COSTS: $14
**Heat management**	• Heatsink 5.2 °C/W • Pre-cut thermal adhesive tape	**All the previously mentioned components**	**All the previously mentioned components**	**–**
**Optics**	• Khatod 10° lens • Fraen 21° lens • Khatod 40° lens	**All the previously mentioned components**	**All the previously mentioned components**	**–**
**Mounting**	• Lens holder • Three-pronged clamp	**All the previously mentioned components AND:** o Stand rod o Double bosshead o Retort stand base, tripod	**All the previously mentioned components**	**Comar fine-tuner stand**^**f**^**:** Basic carrier, pinion stage, post-holder, rod, self-manufactured base. COSTS: ~ $ 280 **Cross-clamp to fixate DC plug on arm**^**g**^**:** COSTS: ~ $12
**Average costs**	**~ $ 65**	**~ $ 90**	**~ $ 128**^**c**^**(Arduino clone: $ 116)**	

^a^ For components originally listed in euros, we calculated the dollar price with the current exchange rate in mind. All prices are excluding soldering material and standard materials such as extra wires and shrink tubing. Also, standard worm equipment is not taken into account (e.g., microscopes and worm trackers). ^b^ The low-cost stand provides a way to work with the WF-NTP and a microscope set-up and is therefore the most flexible. ^c^ The price is based on an original Arduino Uno R3. Clones tend to be much cheaper, but identical in the board lay-out. ^d^ The components are not essential, but potential substitutes or additions. ^e^ When a Tri-Star LED or any other LED is used, we highly recommend to re-evaluate the thermal management. The heatsink of the OptoArm was specifically selected based on the specification of a single royal-blue LED. ^f^ The Comar fine-tuner stand is a more expensive stand, but allows fine adjustments of height due to the pinion stage. All the specifications can be found in the method section. ^g^ We did use the cross-clamp to organize the OptoArm and attach the DC contra plug, see Fig. 2C

**Table 3:** Instructions for building the OptoArm (see Fig. 2C)

Steps	Goal	Instructions^a^	Critical notes
**Step 1**	Connecting power	In order to connect a DC adapter to the optogenetics system, we use a soldering-free DC plug. The black and red wires of the connecting wire harness (I) should be attached to the DC plug (II, III). Note that the used wire harness has already a 5 kΩ potentiometer pre-installed. When using a different system, make sure to manually add a potentiometer parallel to the switch between the yellow (REF) and gray (CTRL) wires.	Determine the center and barrel polarity of the system to ensure appropriate wiring (with a multimeter)
**Step 2**	Connecting LED and thermal management	For appropriate thermal management use a heatsink that fulfills the criteria of the system (see “Thermal management”, Table 1). Clean the heatsink with ethanol before adding a pre-cut thermal adhesive (I). Remove the top-layer of the thermal adhesive and place the LED on top of it (II). Apply firm pressure for at least 30 s with an assembly press that is included with the single rebel led (III). Solder the blue and white wires of the connecting wire harness to the cathode and anode of the LED respectively. Then, cut some pre-cut thermal adhesive in the shape of the lens holder and attach the lens holder via the tape to the heatsink (IV). Finally, by loosening the screw of the lens holder, lenses can be added to or removed from the system (V).	It is important to solder when the LED is already on the heatsink to ensure heat dissipation and circumvent overheating of the LED
**Step 3**	Connecting swtich and LED driver	Add the ON/OFF switch (I) and LED driver (II) to the system (III, IV). The driver can be connected to the wire harness by soldering male pin connectors to the wires of the LED driver and clicking them in the connecting wire harness. Note, that the pictures (Fig. 2C) show the serial set-up used in the paper, in which the red-wire coming from the DC plug is connected with the ON/OFF switch before connecting to the LED driver. We recommend to place the switch in parallel to the potentiometer between the yellow and gray wires (See Fig. 2A).	The placement of the ON/OFF switch between the CTRL and REF pins, in parallel with the potentiometer provides the best management of inrush power and spikes to the LED.
**Step 4**	Mounting on the arm: the final OptoArm	Add the whole circuitry to a standard flask clamp (I). Use a cross-clamp to fixate the DC plug on the arm (II, III). By placing the OptoArm in either a fine-tuner or a general lab standard, the system can be used for different applications.	–

^a^ The different steps and roman numerals refer to those used in Fig. 2C

The resulting OptoArm is compact in nature, making it easy to move around and integrate within different recording systems. The prices in Table 2 are an approximation based on the main components. They do not include the required, basic soldering materials, extra wires, shrink tubing and pin-adaptors, nor standard *C. elegans* equipment, like a microscope*.* We were able to reduce our costs further (< $65) by recycling available material in the lab, including the flask clip clamp and different stands. Therefore, it should be underlined that almost all components of the OptoArm can be substituted for cheaper alternatives that may be readily available. The mounting and optical components (Table 2) can be easily interchanged with alternatives. The required components related to thermal management, however, are highly dependent on the requirements of the electronic components (see “Thermal management” section). Moreover, the choice for the specific LED driver in this paper is also related to the possible automation of the system, which we will touch upon in later sections. In the end, it comes down to the user to verify that any alternative components meet the required specifications of the system and that the system still performs adequately for the desired experimental set-up.

#### Thermal management

Although LEDs rank among the most efficient sources of illumination, a large portion of the input power is still converted to heat [85, 86]. It is important to have adequate thermal management to remove this waste heat by either conduction or convection, as excessive increases in temperature at the LED junction directly affect LED performance. In the short term, this could result in color shifts and reduced light output (intensity), while in the long term accelerated lumen depreciation may take place [87–91]. While the so-called “T-droop”, i.e., the efficiency droop with increasing temperature, is more pronounced in AlGalnP-based LEDs (e.g., red LEDs), blue InGaN-LEDs still suffer from (minimal) color shifts and decreased output due to high junction temperature [92–94]. The junction temperature of a LED is determined by three parameters: drive current, heat dissipation, and the ambient temperature [92]. While the drive current can be kept stable with fixed-current drivers and ambient temperature can be controlled for in climate-controlled rooms, efficient heat removal generally requires extra management. Heat is typically transferred away and dissipated from the LED using passive systems like heatsinks (Fig. 2F). The required thermal properties of such a heatsink can be calculated by using a basic thermal model: see Table 4. We selected a 5.2 °C/W heatsink that fulfills our criteria for a system with appropriate thermal cooling. It is important to underline that the measured junction temperature is higher than theoretically expected when using a heatsink of 5.2 °C/W (see Table 4). Therefore, it is critical to use a safe margin when selecting a heatsink and to verify the actual junction temperature instead of simply relying on the thermal equation.

**Table 4:** Calculating the required thermal properties of a heatsink

Using the following thermal model (eq. 1, [85], Fig. 2F) and calculating or estimating all the individual parameters (eq. 2–4), the required thermal properties of a heatsink can be calculated.	
$$ {R\theta}_{J-\mathrm{Ref}}=\frac{\Delta {T}_{J-\mathrm{Ref}}}{P_D} $$	(1)
Where:	
$$ {\displaystyle \begin{array}{c}{R\theta}_{J-\mathrm{Ref}}=\mathrm{Thermal}\ \mathrm{resistance}\ \left({}^{\circ}\mathrm{C}/\mathrm{W}\right)\ \mathrm{from}\ \mathrm{the}\ \mathrm{LED}\ \mathrm{junction}\ \mathrm{to}\ \mathrm{a}\ \mathrm{reference}\ \mathrm{point}\\ {}={R\theta}_{\mathrm{Junction}-\mathrm{Thermalpad}}+{R\theta}_{\mathrm{Thermalpad}-\mathrm{Solderpad}}+{R\theta}_{\mathrm{Solderpad}-\mathrm{Adhesives}}+{R\theta}_{\mathrm{Adhesive}-\mathrm{heatsink}}\end{array}} $$	(2)
∆*T*_*J* − *Ref*_ = (TJ, junction temperature) – (TRef, reference point temperature, °C)	(3)
$$ {\displaystyle \begin{array}{c}\mathrm{PD}=\mathrm{Power}\ \mathrm{dissipation}\ \left(\mathrm{W}\right)\\ {}=\mathrm{LED}\ \mathrm{forward}\ \mathrm{current}\ \left(\mathrm{If}\right)\ast \mathrm{LED}\ \mathrm{forward}\ \mathrm{voltage}\ \left(\mathrm{Vf}\right)\end{array}} $$	(4)
Typically, the maximal junction temperature can be found in the datasheet for the LED. For a Royal-Blue Luxeon Rebel LED on a SinkPAD-II that is used in this paper, the listed temperature is 150 °C. Ideally, the operating temperature should be well below that limit, as even reaching this temperature for a fraction of time could influence the properties of the LED [92–95]. Therefore, we decided to set the maximum junction temperature to 100 °C. Next, the power dissipation of the same LED can be calculated by multiplying the forward voltage rating of the LED with the drive current in Amperes (eq. 4). Since we use a fixed 700-mA LED driver and know the maximal voltage drop of the LED (3.5 V), P_d_ is easily calculated (eq. 4). Finally, the maximal thermal resistance should be calculated by taking in the maximum working ambient temperature into account, which was determined to be 25 °C (20 °C room temperature + 5 °C margin, eq. 3). Importantly, note that our set-up is located in a climate-controlled room. If this is not the case, a wider margin for temperature fluctuations and higher maximum temperature based on local environmental conditions should be considered. Rewriting eq. 1 and calculating *Rθ* gives (eq. 5):	
$$ {R\theta}_{J-\mathrm{Ref}}=\frac{\Delta {T}_{J-\mathrm{Ref}}}{P_D}=\frac{\Delta {T}_{J-\mathrm{Ref}}}{If\ x\ Vf}=\frac{100-25}{0.7\times 3.5}=30.6{}^{\circ}\mathrm{C}/\mathrm{W} $$	(5)
This means that the total thermal resistance of the system can be maximally 30.6 °C/W (eq. 2) when one aims for junction temperatures that are maximally 100 °C. In order to see what that means for the required heatsink, all the known thermal resistances (Fig. 2F) can be collected from the datasheets of the LED (junction to thermal pad: 6.0 °C/W and thermal pad to solder pad: 0.7 °C/W) and used adhesives (4.5 °C/W). Rewriting eq. (2) gives (eq. 6):	
$$ {\displaystyle \begin{array}{c}{R\theta}_{\mathrm{Adhesive}-\mathrm{Heatsink}}={R\theta}_{J-\mathrm{Ref}}-{R\theta}_{\mathrm{Junction}-\mathrm{Thermalpad}}-{R\theta}_{\mathrm{Thermalpad}-\mathrm{Solderpad}}-{R\theta}_{\mathrm{Solderpad}-\mathrm{Adhesives}}\\ {}=30.6-6.0-0.7-4.5\\ {}=19.4{}^{\circ}\mathrm{C}/\mathrm{W}\end{array}} $$	(6)
Theoretically, a heatsink in this set-up can have a maximal thermal resistance of 19.4 °C/W and any heatsink with lower resistance than that can be used. We used a finned heatsink with thermal resistance of 5.2 °C/W to keep the set-up compact and still achieve sufficient cooling. Next, we continued with an empirical approach to verify whether appropriate heat dissipation could be obtained with this set-up. Therefore, we measured the actual junction temperature and the voltage drop of the LED mounted to the OptoArm (Fig. 2D). With a Fluke TM80 module and associated test probe, we measured the temperature at the specified test location (Fig. 2F) of the LED for about 1 min until the temperature stabilized. At the same temperature, we also determined the actual voltage drop of the LED. We measured a temperature of 55.68 ± 0.76 °C (*n = 10*) and a forward drop of 2.972 ± 0.004 V (*n = 5*). By using eq. (7), the actual junction temperature can be derived:	
$$ {\displaystyle \begin{array}{c}{T}_{\mathrm{Junction}}={T}_{\mathrm{testpoint}}+\left({R\theta}_{\mathrm{Junction}-\mathrm{Thermalpad}}+{R\theta}_{\mathrm{Thermalpad}-\mathrm{testpoint}}\right)\times \left({V}_f\times {I}_f\right)\\ {}=55.68{}^{\circ}\mathrm{C}+\left(6.0{}^{\circ}\mathrm{C}/\mathrm{W}+0.5{}^{\circ}\mathrm{C}/\mathrm{W}\right)\times \left(2.972\ V\times 0.7\ A\right)=69.2{}^{\circ}\mathrm{C}\end{array}} $$	(7)
With an estimated junction temperature of 69.2 °C, the system fulfills our criteria for a system with appropriate thermal cooling. This is further evidence by the temperature measured after 10 min of constant illumination at the maximal capacity: 55.81 ± 1.09 °C (=junction temperature of 69.3 °C, *n = 10*).	

### Lighting area and intensity is stable and can be adjusted to experimental requirements through exchangeable lenses and adjustable working distance

**Fig. 3:**
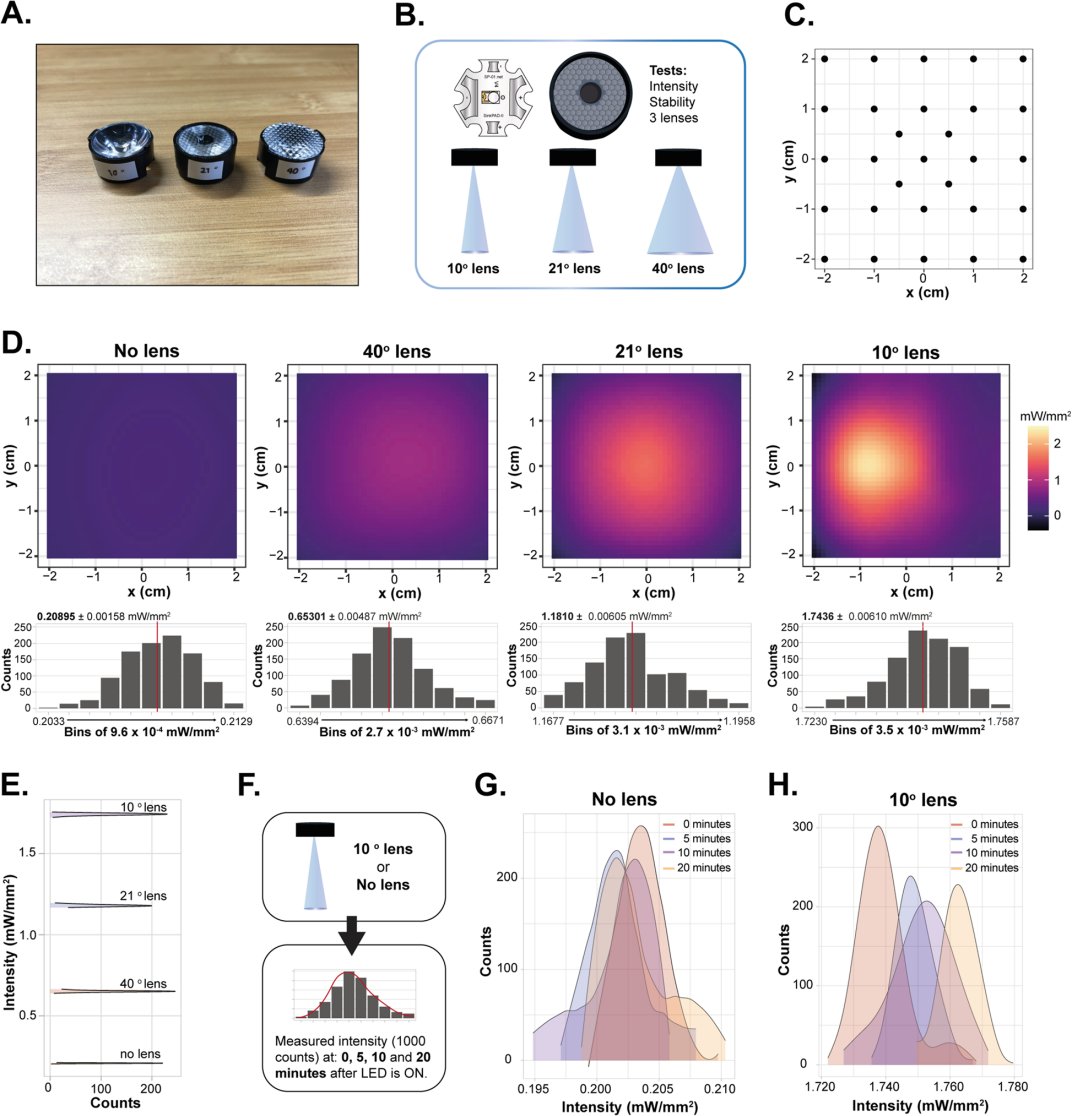
The OptoArm provides the light intensity and stability required for optogenetic experiments. **A** The different lenses used for testing. **B** A schematic outlining the tested parameters with the three different lenses. **C** Raster showing the *x,y* coordinates used to measure the intensity of the LED. **D** The different integrated intensity profiles of the LED with and without lenses. The histograms show the zoomed-in distribution of intensity readings between a minimum and maximum intensity with a specified bin width. Red lines show the average intensity. **E** Smoothened histograms of **D** that show the increase in intensity when using different lenses with the OptoArm. **F** Schematic showing the method to assess the stability of intensity over time. **G** Distributions of intensity readings at the different timepoint without a lens (**H**) and with a 10° lens. All readings were performed at 448 nm

Next, we explored the usability of the OptoArm for experimental purposes with *C. elegans*. We evaluated different aspects of the system that are critical for optogenetics, starting with light intensity and stability. The Royal-Blue Luxeon Rebel LED of the OptoArm has a beam angle of 125°, which results in a large surface being illuminated. As intensity is the function of power divided by surface, there are simply two parameters that determine the intensity of a system: (1) area being illuminated, (2) the power of the LED. The maximal power of the LED (1.03 W) at a current of 700 mA is fixed and cannot be further enhanced. However, the area that is illuminated can be reduced by either decreasing the working distance (WD) or applying convex optics to concentrate the light beam to a smaller surface. Since optogenetic experiments in *C. elegans* require an intensity of at least 1.0 mW/mm^2^ and preferably 1.6 mW/mm^2^ [31–33, 62], we explored the produced intensity of the OptoArm without and with several low-cost lenses (Fig. 3A, B).

To compare the different set-ups, we used a fixed working distance of 5 cm between the LED and the surface to be illuminated. With a power meter set to 448 nm, we measured the light intensity of 29 different *x,y* coordinates in an area of 4 by 4 cm using a “hot-spot” approach (Fig. 3C). From those collected measurements, we generated integrated intensity profiles (Fig. 3D). We observed clear differences in the acquired intensity profiles between the different set-ups. Without a lens, the illumination is relatively uniform, while using lenses that narrow the angle of the light beam result in more pronounced Gaussian-like profiles with clear intensity peaks in the middle. The combination of the OptoArm with a 10° lens fulfills the required intensity of 1.6 mW/mm^2^. In order to assess the stability of these profiles, we collected 1000 (~ 1 min) readings per set-up at the *x,y* coordinate with the highest intensity, thereby assuming an Gaussian-distributed light source. For all the set-ups, the readings were steady and consistent, as evidenced by the narrow distributions (Fig. 3D, E). Clearly, adding different convex lenses to the OptoArm can modify the output of the system in terms of measured light intensity (Fig. 3E). Note that the readings in the histograms (Fig. 3D, E) are lower than those seen in the intensity profiles (Fig. 3D). The latter can be explained by the different measuring methods (software-related) that were used: hot-spot detection versus a Gaussian assumption.

Although most optogenetic experiments only take a few milliseconds, or a minute at its maximum [84], we also assessed the stability of the system in terms of intensity over several minutes (Fig. 3F). With the same working distance of 5 cm, we assessed the maximum intensity at 0, 5, 10, and 20 min after turning the LED on. Again, we collected 1000 readings per timepoint. The ON time of the LED had no influence on the measured intensity without a lens (Fig. 3G), while only a minor drift in intensity (< 0.02 mW/mm^2^ in 20 min) was observed with the 10° lens (Fig. 3H). Therefore, we conclude that the OptoArm, in its current set-up, performs well in terms of intensity and stable output over time.

### Due to its compact and highly flexible nature, the OptoArm is compatible with both single- and multi-worm imaging platforms

**Fig. 4:**
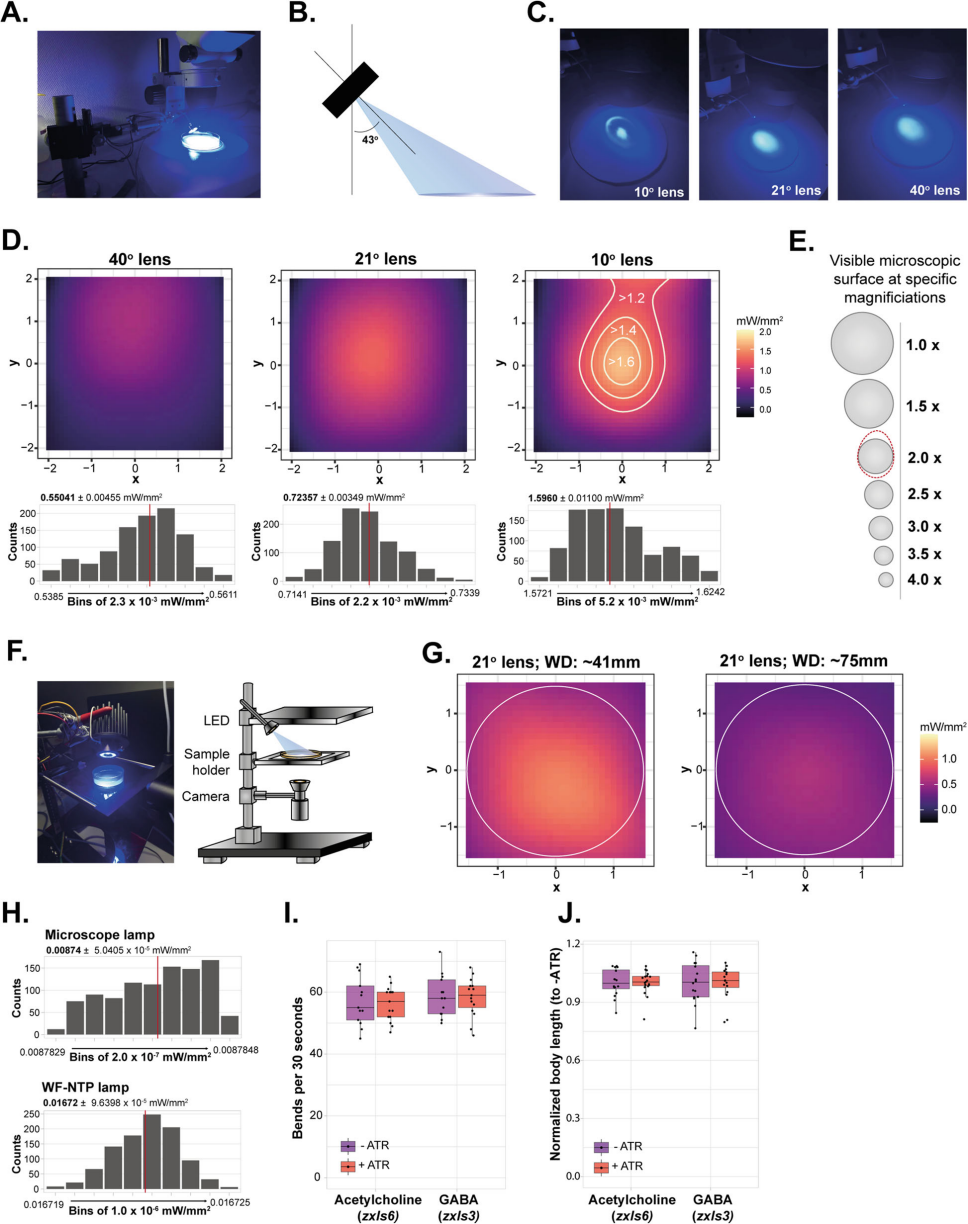
The OptoArm fulfills all technical criteria in different experimental set-ups. **A** The OptoArm set-up used with a standard dissection microscope. **B** In experimental set-ups, the OptoArm is used with a specific angle (43°), thereby deviating from perpendicular illumination. **C** The different illumination profiles for the different lenses upon and illumination with the specified angle of incidence. **D** The different integrated intensity profiles for **C**. The histograms show the distribution of intensity readings between a minimum and maximum intensity with a specified bin width. Red lines show the average intensity. **E** Schematic showing the different visible microscopic areas (of the used microscope) when using different magnifications. The areas are on scale and can be directly compared to the intensity maps shown in **D**. 1.0× corresponds to a total magnification of 15×, and 4.0 to a total of 60×. The dotted red line represents the area in **D** with an intensity of > 1.6 mW/mm^2^. **F** The OptoArm set-up with the WF-NTP. **G** The different integrated intensity profiles of the LED with the 21° lens, at different working distances with a fixed angle of incidence of 43°. Profiles were generated in the set-up with the WF-NTP. Circles represent the outline of a 3-cm NGM plate. **H** Histograms of intensity readings of the microscope-light (Gaussian) and WF-NTP backlight (flat-field). Red lines show the average intensity. All readings were performed at 448 nm. **I** Thrashing frequency of D1 worms, expressing ChR2 under the *unc-17* (acetylcholine) or *unc-47* (GABA) promotor, recorded with the WF-NTP, grown with or without ATR. There is no significant effect of the WF-NTP light on this behavior, *n = 15*, two-tailed unpaired Student’s *t* test (all: n.s.). **J** Body length of D1 worms recorded with the WF-NTP, grown with or without ATR. Body length was normalized by the mean of the paired ATR− condition. There is no significant effect of the WF-NTP light on this behavior, *n = 15*, two-tailed unpaired Student’s *t* test (all: n.s.). For **I** and **J**, there was no photo-stimulation with the OptoArm

When using the OptoArm with a microscope or any tracking platform (e.g., the wide field-of-view nematode tracking platform (WF-NTP) [82, 83], the angle of incidence of the blue light is in most cases not perpendicular to the surface as a consequence of practical limitations, e.g., the arm should not block the light-pad from the sample to the ocular or camera (Fig. 4A). The consequence of working under a non-perpendicular angle is that intensity profiles will deform due to increases and decreases of the path length of specific parts of the light beam (Fig. 4B, C). The intensity of light as a function of the distance from the light source follows an inverse square relationship. Therefore, increased path lengths will result in a significant reduction of the light intensity.

**Fig. 5:**
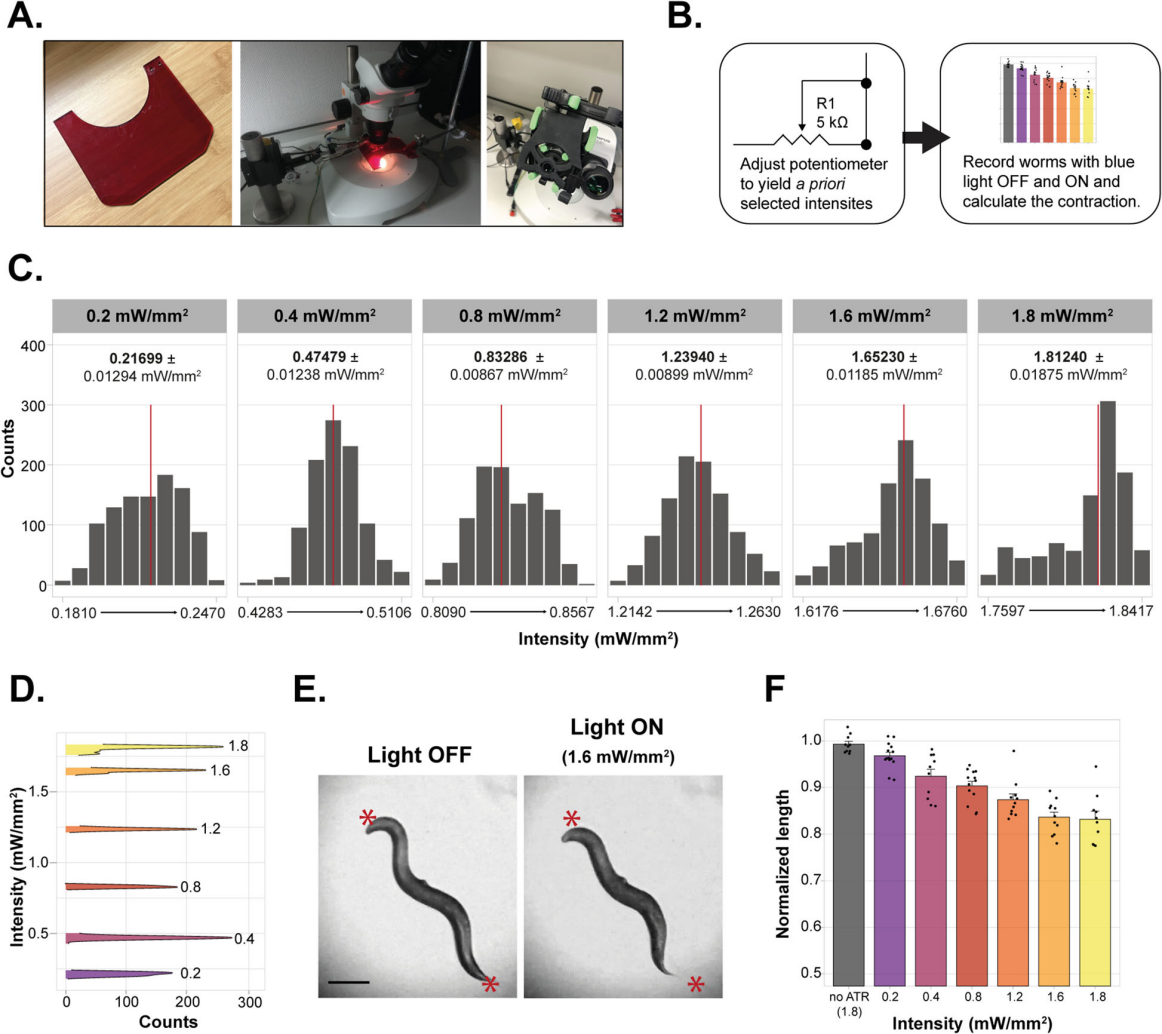
The OptoArm allows manual intensity adjustments, which modulate biological readouts. **A** To record worms in an inexpensive way, we used a Carson smartphone adapter to place and orient a cellphone camera to a microscope. A blue light filter was placed between the ocular and sample to avoid interference of the blue light with recording. **B** Schematic of the experimental outline: a priori determined intensities (0.2, 0.4, 0.8., 1.2., 1.6, and 1.8 mW/mm^2^) were set by adjusting the resistance of the potentiometer. At the different intensities, the ∆body length was measured**. C** The histograms show the distribution of intensity readings at a priori-specified intensities. Red lines show the average intensity. **D** Smoothened histograms of **C** that show the increase in intensity when adjusting the resistance of the potentiometer. **E** Pictures of a D1 worms (*myo-3p::ChR2::YFP*) just before and during illumination with 1.6 mW/mm^2^ blue light. Scale bar: 200 μm. **F** The different body lengths (normalized to length before illumination) at different intensities of illumination with the OptoArm. Calculated via a midline approach, *n =* 10

To validate the OptoArm in an actual experimental set-up, we again generated integrated intensity profiles and stability measurements of the three lenses when used with a standard dissection microscope (Fig. 4). As expected, both the hot-spot intensity and the steady-state measurements pointed out that the maximal intensity is decreased when working under a non-perpendicular incidence angle (Fig. 4D). However, the acquired intensity with a 10° lens still reaches the required 1.6 mW/mm^2^ (WD: ~ 3.5 cm) and with some fine adjustments in height (i.e., working distance) and angle, the intensity even exceeds this number (see also Fig. 5). More importantly, the area with the required intensity (1.6 mW/mm^2^) is large enough to ensure the whole visible microscopic surface is being illuminated with the same intensity (see Fig. 4E for details). In fact, an area with a ~*ϕ* 1 cm appears to fulfill the required 1.6 mW/mm2 (Fig. 4D) and this area corresponds with the visible microscopic surface at a total magnification of × 30 (Fig. 4E, × 2.0).

Having established and validated a microscope set-up, we moved on to an experimental set-up involving a multi-worm tracker (Fig. 4F). When working with a platform that allows multiple worms to be tracked at the same time, it is critical that illumination and light intensity are as uniform as possible at all *x,y* coordinates in the region of interest (ROI). Spatial consistency is relevant in order to reduce individual variation. Clearly, lenses that generate narrower Gaussian-like profiles (Fig. 4D) do not meet those criteria, even though they do provide the adequate intensity. Consequently, we only generated intensity profiles of the OptoArm with the 21° lens at two different working distances (Fig. 4G) and a fixed incidence angle of 43°. While far from perfect, the 21° lens offers the best compromise between uniform illumination and high intensity (0.75–1.0 mW/mm^2^) when using 3-cm worm plates (Fig. 4G). Moreover, systems like the WF-NTP [82, 83] make it possible to only analyze worms in a specific ROI, which would make it possible to account for spatial inconsistency of illumination at the edges of a plate. If the experimental conditions allow it, one can also simply use smaller plates, e.g., 12 wells, to better fit the intensity profiles. Finally, it should be underlined that the adaptability of the OptoArm makes it easy to further enhance the system, e.g., by using triple-LED systems instead of single LEDs to triple the power of the system (Table 2) if required.

Finally, we assessed the intensity of backlights of a standard microscope (maximal intensity) and the WF-NTP (Fig. 4H). While the intensity of those lights is relatively low, it might be sufficient to induce optogenetic responses [35]. We have tested this hypothesis by comparing the thrashing capacity (i.e., the frequency of lateral bends in liquid) and body length of worms grown with or without ATR with the WF-NTP. Since ATR is required for functional ChR2, non-ATR worms will not respond to blue light and can be used as negative controls [32]. We did not find clear evidence for a light-induced response when performing optogenetic experiments with the WF-NTP backlight. Worms grown with or without ATR had a similar thrashing capacity and body length (Fig. 4I, J). However, we would recommend to substitute the WF-NTP backlight with a red-shifted variant of 625 nm (Edmund Optics, Red advanced illumination side-fired backlight, #88-411) or to filter out the blue light specifically. This is advisable because several neuronal mutants have been shown to have an increased sensitivity to low-light conditions [33]. For microscope set-ups, we suggest to turn down the backlight as much as possible, without compromising the contrast between the worm and its background (Fig. 4H).

### Adjustable intensity levels enable titration of the biological response

All *C. elegans* neurons that have previously been analyzed by photo-electrophysiology exhibited graded transmission. Herein, the release of neurotransmitters directly correlates with the extent of depolarization evoked by light-induced ChR2 activation [31]. This type of transmission has clear consequences for optogenetic experiments, as changing the light intensity will likely also change transmitter release. Therefore, to properly compare different interventions or different mutants with each other, light intensity should always be consistent and stable throughout complete experiments. On the other hand, the graded transmission in *C. elegans* also allows the “titration” of the extent of ChR2-induced depolarization by adjustment of the light intensity. In this way, one can modify transmitter release and subsequently also fine-tune the behavioral output [33, 60].

We explored the adjustability of the OptoArm for “titration” purposes (Fig. 5). First, we created a basic microscope set-up in which a microscope-based smartphone adapter was used to allow movies being made with a smartphone instead of an expensive camera (Fig. 5A). This set-up was completed with a standard blue light/UV protection shield to avoid saturation of the camera. Secondly, we used the potentiometer of the OptoArm to adjust the intensity of the system to specific values, without changing the WD or angle of incidence (e.g., 0.2, 0.4, 0.8, 1.2, 1.6, and 1.8 mW/mm^2^) (Fig. 5B). Note that the previously established intensity maps were used for validation of the OptoArm; for experimental purposes, we normally only measure the area of interest (e.g., the microscopic area or the ROI of the WF-NTP). Then, we measured both the stability of each specified light intensity and the accompanying body length of worms expressing ChR2 under the *myo-3* promoter before and during illumination (Fig. 5C–F). The normalized body length was then calculated by dividing the body length during illumination by the body length before illumination. We were able to adjust the potentiometer in such a way that the a priori-specified intensities could be acquired relatively well and were consistently stable over 1000 readings (Fig. 5C, D). Clearly, adjusting the resistance of the potentiometer of the OptoArm can modify the output of the system in terms of measured light intensity (Fig. 5D). The different light intensities had dose-dependent effects on normalized body length up till 1.6 mW/mm^2^ (Fig. 5E,F). Taken all together, the OptoArm allows users to change the light intensity and thus to study its effect on behavioral changes.

### Perimeter and midline measurements are highly correlated and can be used interchangeably as a readout for body length

**Fig. 6:**
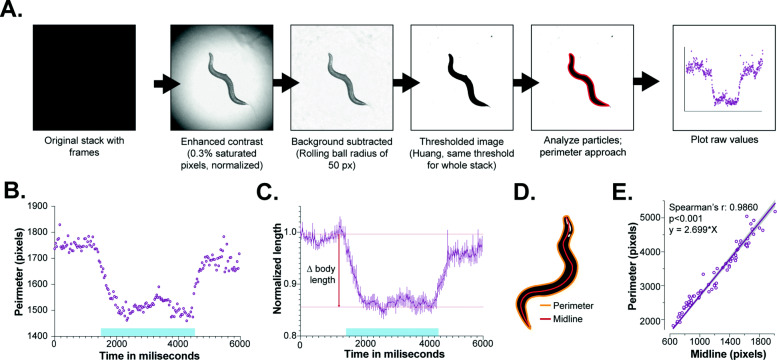
A semi-automatic perimeter approach is a reliable way of assessing changes in body length during and after optogenetic stimulation. **A** Schematic outline of image processing in Fiji to follow body length changes over time in a semi-automatic way. **B** Raw readings of body length (perimeter in pixels) of a worm expressing ChR2 in cholinergic neurons (*unc-17p::ChR2::YFP*) plotted against time in milliseconds, the graph represents *n* = 1. **C** The change in body lengths (normalized to length before illumination) of multiple worms, *n = 10*, when using a perimeter approach to estimate the worm length. ∆body length equals the change in length before illumination and during illumination. **D** Schematic of a worm with the midline and perimeter highlighted. **E** The relationship between the midline of the worm and the perimeter. *n = 70* (35 still images of worms at light ON and 35 worms at light OFF). The spearman correlation was calculated (*p* < 0.001)

Before continuing with the biological validation of the OptoArm, we first took the time to explore the possible methods to quantify changes in body length during an optogenetic experiment. In general, to follow changes in body length over time, one can manually draw a midline through the worm from head to tail before and during blue light stimulation (Fig. 5). Clearly, this labor-intensive scoring method is vulnerable to inconsistency and experimenter bias and becomes impractical when scoring many worms and conditions. There are Matlab codes available to automate and streamline these measurements [33, 74], but these codes also require a basic understanding of programming languages and access to Matlab. Based on the idea that the OptoArm should be a simple system that is accessible to the whole community, we aimed to simplify the accompanying analysis for “∆body length,” i.e., the change in body length upon optogenetic stimulation, as well. Therefore, we explored the use of the open-source software Fiji [96] to analyze the optogenetic data in a semi-automatic way (Fig. 6).

It has been stated that the perimeter of the worm is equal to two times the body length plus two times the diameter [97]. Since the perimeter can be easily measured and is more often used as a way to represent body length in *C. elegans* [97–99], we created a sequential pipeline in Fiji [96] with the perimeter as readout (Fig. 6A). All steps of this Fiji pipeline, and the accompanied methods, are included in the “Methods” section. In short, the pipeline focusses on enhancing contrast, subtracting background and thresholding to yield a set of binary images from the worm without gaps or ruffled edges. The binary images are used to measure the perimeter of the worm in each frame. Since all steps are performed on complete movies, the end result is a list of perimeters for a single worm over time. In Fig. 6B, the raw perimeter measurements of a worm expressing ChR2 under the *myo-3* promoter, before, during, and after optogenetic stimulation are plotted. By normalizing all individual measurements of a single worm to the average perimeter in the first 60 frames (light OFF), one can create kinetic plots of the change in perimeter that represent multiple worms (Fig. 6C). In order to validate this method, we measured the length of the midline and the accompanying perimeter of 70 individual worms (Fig. 6D). The midline and perimeter correlate significantly and therefore appear to be both good estimates of body length (Fig. 6E).

### Optogenetic stimulation of cholinergic and GABAergic mutants with the OptoArm accurately reproduces the biological response observed with a more expensive system

**Fig. 7:**
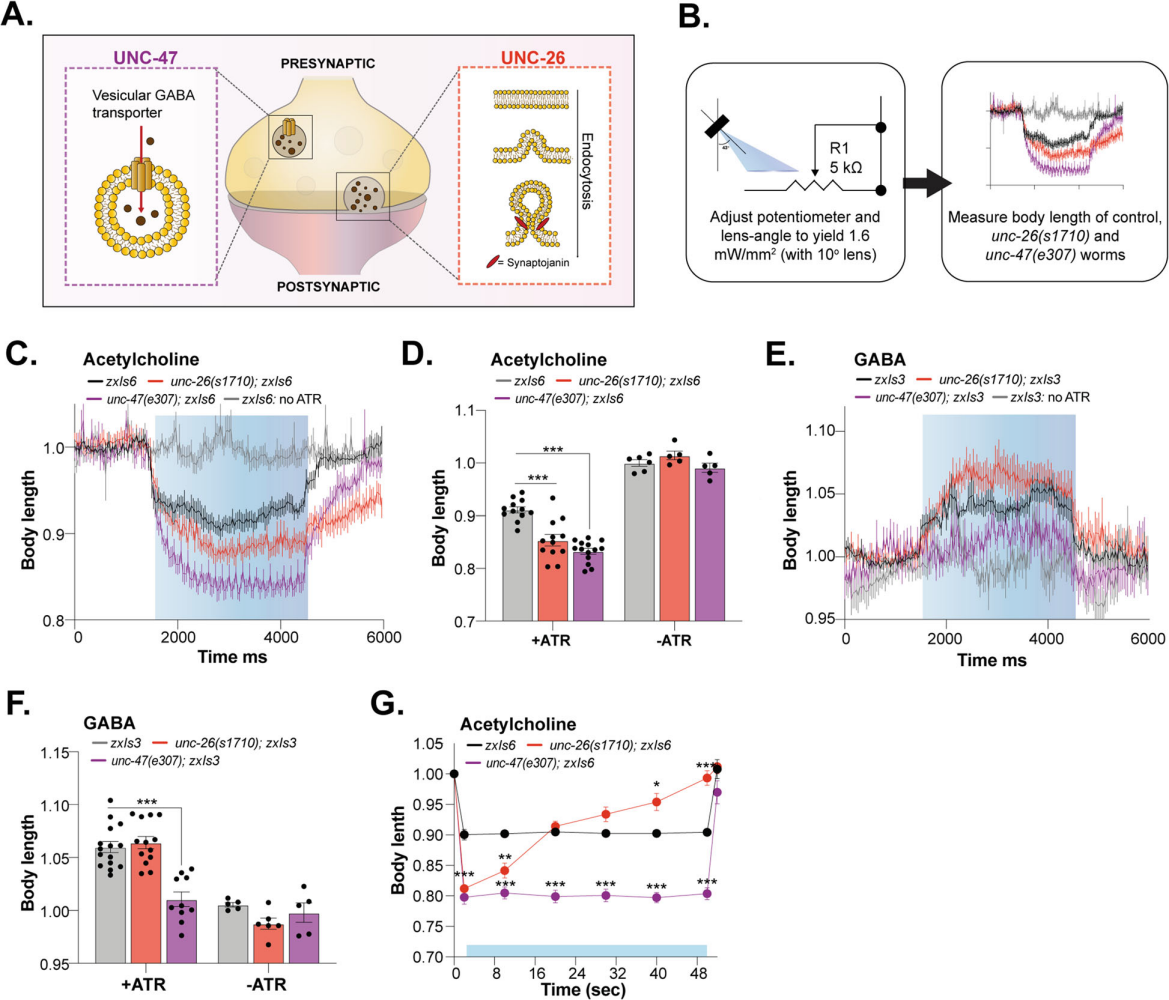
Mutations in the cholinergic or GABAergic system affect ∆body length after optogenetic stimulation with the OptoArm. **A** A schematic showing the different mutant used to verify previously established results. **B** Schematic of the experimental outline: the change in body length was measured after blue light stimulation with an intensity of 1.6 mW/mm^2^. **C** Kinetics of optogenetic stimulation in mean ± SEM body length of control worms and *unc-26(s1710*) and *unc-47(e307)* mutants expressing ChR2 in cholinergic neurons, *n = 9–10*. A perimeter approach was used. **D** Quantification of mean ± SEM normalized body length of control D1 worms and *unc-26(s1710)* and *unc-47(e307)* mutants expressing ChR2 in cholinergic neurons. Two-way ANOVA (Interaction, ATR, Genotype: *p* < 0.001) with post hoc Dunnett, *n = 11–15* for ATR+ and *n = 5–6* for ATR−. A midline approach was used. **E** Kinetics of optogenetic stimulation in mean ± SEM normalized body length of control worms and *unc-26(s1710)* and *unc-47(e307)* mutants expressing ChR2 in GABAergic neurons, *n = 9-11.* A perimeter approach was used. **F** Quantification of mean ± SEM normalized body length of control worms and *unc-26(s1710)* and *unc-47(e307)* mutants expressing ChR2 in GABAergic neurons. Two-way ANOVA (Interaction, ATR, Genotype: *p* < 0.001) with post hoc Dunnett, *n = 10–15* for ATR+ and *n = 5–6* for ATR−. A midline approach was used. **G** Long-term photo-stimulation of control worms, *unc-26(s1710)* and *unc-47(e307)* mutants expressing ChR2 in cholinergic neurons. Two-way repeated ANOVA with Geisser-Greenhouse correction (time, genotype, time × genotype and individual worms: *p* < 0.001) and post hoc Dunnett, *n = 10*. A midline approach was used. Blue bars represent “light ON.” Acetylcholine: *zxIs6 (Punc-17::ChR2::YFP), zxIs3 (Punc-47::ChR2::YFP).* *: *p* ≤ 0.05, **: *p* ≤ 0.01, ***: *p* ≤ 0.001. Error bars represent S.E.M

To validate the OptoArm and accompanying image processing biologically, we aimed to reproduce previously described and published optogenetic data on neuronal mutants (Fig. 7A). It has been showed that mutations in the *unc-47* gene, which represents the vesicular GABA transporter vGAT [100], translate into the lack of body wall relaxation upon optogenetic stimulation of GABAergic neurons, but increased contractions when cholinergic neurons were photo-stimulated [33]. At the same time, mutations affecting the cholinergic system, e.g., *unc-26* that represents the phospholipid phosphatase synaptojanin (Fig. 7A), often translate into significantly stronger body wall muscle contractions upon cholinergic stimulation as well. The latter was initially marked as contradictory as electrophysiological data of those mutants showed reduced electrically evoked postsynaptic currents (ePSCs). However, the paradoxical results are most likely a result of compensatory mechanisms in the muscle, underlining the complementary information that behavioral optogenetic experiments can provide [33].

Here, we explored two mutants described previously, namely *unc-47(e307)* and *unc-26(s1710)* (Liewald et al. 2008). We used a microscopic set-up (Fig. 5A) combined with the OptoArm, thereby ensuring a light intensity of 1.6 mW/mm^2^ (Fig. 7B). Next, D1 adults expressing ChR2 specifically under the *unc-17* promoter (i.e., expression in cholinergic neurons; *zxIs6*), grown on ATR− or ATR+ plates, were exposed to a regime of 3 s light off–3 s light on–3 s light off while being recorded. Subsequently, the movies were used to determine the body length over time by a perimeter approach (Fig. 7C) or by measuring the length at fixed timepoints before (500 ms before light ON) and during illumination (1000 ms after light turned ON) with a midline approach (Fig. 7D). Similar to the results described by Liewald et al., we found that *unc-47(e307)* and *unc-26(s1710)* mutants showed significantly stronger photo-evoked contractions (Fig. 7C), with clear differences in muscle-relaxation kinetics after the photo-stimulation was terminated. In addition, worms raised at ATR− plates, did not respond to blue light with body contractions and served as appropriate negative controls. The change in body length of the *unc-47* (mean ±*SD*:0.83 ± 0.020), and *unc-26* mutants (0.85 ± 0.037) and control (0.91 ± 0.02) worms, as a result of photo-stimulation with the OptoArm, are similar to the numbers found by Liewald et al. (WT: ~ 0.92, *unc-26*: ~ 0.87, *unc-47*: ~ 0.86) [33] (Fig. 7D). Next, we also explored the effects of GABAergic stimulation in the *unc-47(e307*) and *unc-26(s1710)* mutants, by expressing ChR2 under the *unc-47* promotor (*zxIs3*). The same lightening regime was used and the body length was again determined by a perimeter approach (Fig. 7E) and a midline approach (Fig. 7F). As expected, *unc-47(e307)* mutants (1.01 ±0.022) lacked a clear relaxation response, while *unc-26(s1710)* worms (1.06 ±0.020) behaved much more similar to control worms (1.06 ±0.020), with the exception of a higher initial peak of relaxation (Fig. 7E, F). The same observations were described by Liewald et al., (WT: ~ 1.04, *unc-26*: ~ 1.00, *unc-47*: ~ 1.04) [33].

It has been shown that characteristic defects in neurotransmitter recycling can be reflected by aberrations in long-term photo-stimulation experiments with worms expressing ChR2 under the *unc-17* promoter. Synaptojanin (*unc-26*) is required for endocytotic recycling of synaptic vesicles and mutants are defective in synaptic vesicle budding and uncoating during clathrin-mediated endocytosis (Fig. 7A) [101, 102]. The inability to recycle vesicles results in early fatigue of neurons that could potentially translate in the inability to sustain contraction of body wall muscles. Indeed, Liewald et al. [33] described that wild-type worms normally sustain constant contraction throughout long-term illumination (> 60 s), while the initially exaggerated body contraction of *unc-26(s1710)* mutants returns quickly to unstimulated levels. We validated this biological data with the OptoArm by following individual worms for 52 s, in which worms were constantly illuminated from 1 to 51 s (Fig. 7G). While control worms and *unc-47(e307)* mutants showed sustained contraction over the complete interval, *unc-26(s1710)* mutants lost their contractibility already after 8 s and returned to wild-type levels after 15 s. At the end of the illumination period, the *unc-26(s1710)* worms returned to their initial non-illuminated length and thus lost their contraction completely (Fig. 7G). Altogether, we validated the OptoArm by showing that the inexpensive system can reproduce biological findings acquired with a more expensive fluorescence microscope. The use of the OptoArm with Fiji [96] provides a sensitive platform to study the various functional aspects related to body contraction, elongation, and the kinetics of this behavior.

### Combination of the OptoArm with various set-ups allows the readout of multiple biological parameters for a more comprehensive analysis

Having a technically and biologically validated system, we continued exploring the use of the OptoArm for different applications and set-ups. Since the platform also allows multi-worm illumination (Fig. 4), we explored the use of the OptoArm together with a multi-worm tracking platform, in this case the WF-NTP [82, 83]. It has been previously shown that photo-stimulation of worms on solid substrates, expressing ChR2 under the *unc-47* promotor (GABAergic neurons) or under the *unc-17 promotor*, reduces locomotion speed [33, 50]. The observed decline in locomotion speed is caused by different mechanisms. The photoactivation of ChR2 in GABAergic neurons induces a flaccid paralysis, during which worms straighten up completely for a few seconds [33]. This observation is consistent with the inhibitory role of GABA in the neuromuscular system. Stimulation of cholinergic neurons, on the other hand, causes a spastic paralysis during which worms display dorsal coiling behavior in addition to body wall muscle contraction [33, 50]. This coiling behavior can be quantified by following changes in eccentricity. Eccentricity is used as a measure of how nearly circular an ellipse is. Typically, crawling and thrashing worms have an average eccentricity close to 0.93 or higher. At the same time, extreme coilers can have an average eccentricity close to 0.6–0.7 [83].

**Fig. 8:**
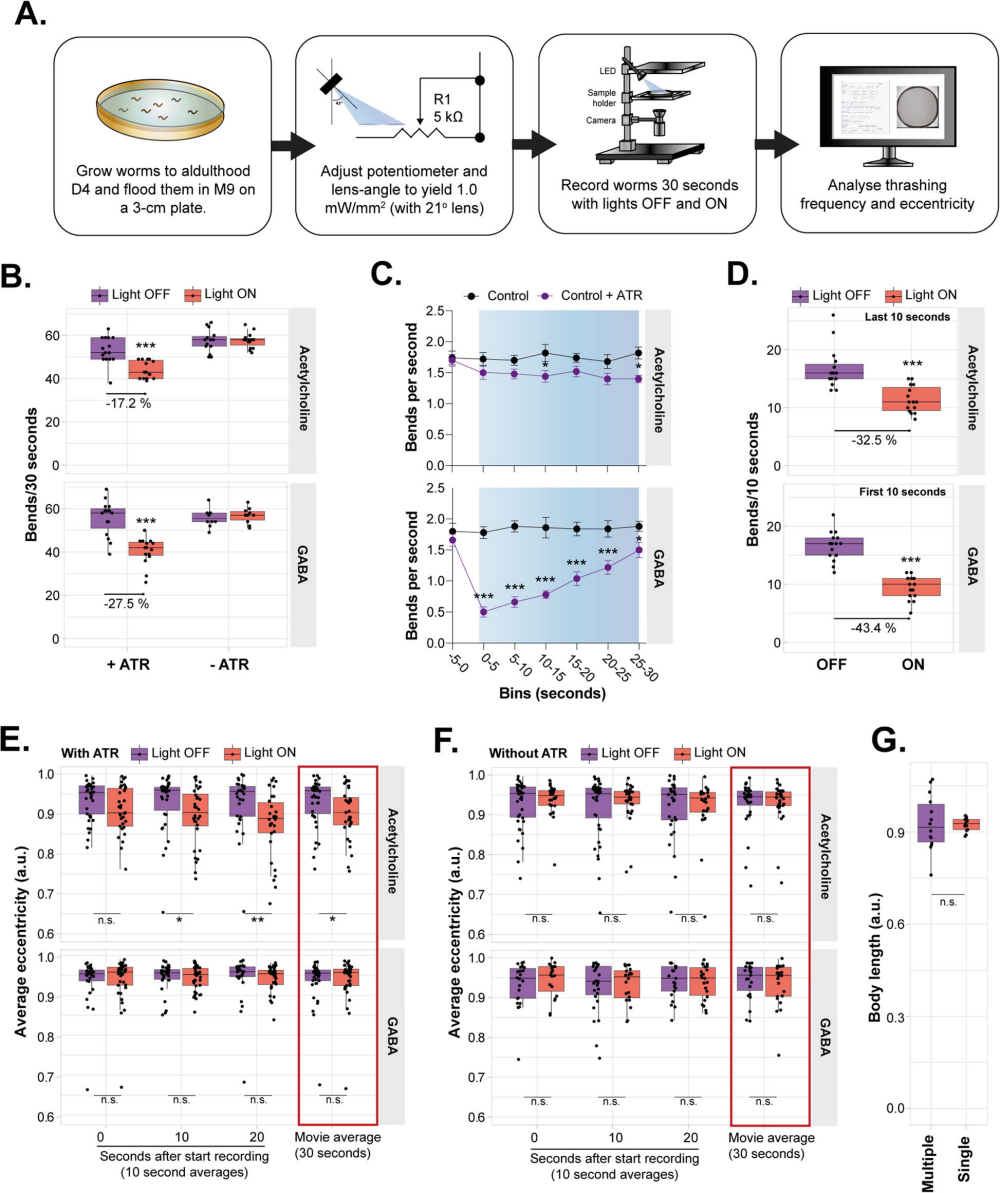
Population characteristics acquired with the WF-NTP are potential readouts of optogenetic stimulation with the OptoArm. **A** Schematic of experimental outline. D4 worms were assessed in liquid with the OptoArm being OFF and ON and population characteristics (bending frequency and eccentricity) were analyzed with the WF-NTP software. **B** Change in bends per 30 s when light is ON of worms grown with or without ATR. The percentual decline in thrashing capacity is annotated in the graph, *n = 15.* For acetylcholine: Mann-Whitney *U* test *p* < 0.001, GABA: two-tailed unpaired Student’s *t* test: *p* < 0.001. **C** Binned effect of blue light on swimming behavior of transgenic worms grown with or without ATR, *n = 10*. Two-way ANOVA (time, genotype, interaction: *p* < 0.001) with post hoc Sidak’s. **D** Change in bends per 10 s after optogenetic stimulation. Acetylcholine: only the last 10 s of 30-s illumination is used, GABA: only the first 10 s are used. *n = 15.* For acetylcholine: Mann-Whitney *U* test *p* < 0.001, GABA: two-tailed unpaired Student’s *t* test: *p* < 0.001. **E** Effect of blue light on eccentricity of worms grown with or **F** without ATR. *n = 20–40*, Mann-Whitney *U* tests acetylcholine + ATR: 0–10 s: not significant, 10–20 s: *p* = 0.0192, 20–30 s: *p* = 0.0033, for 0–30 s: *p* = 0.0427. Acetylcholine—ATR, and both GABA conditions: n.s. **G** The body length of worms expressing ChR2 under the *unc-17* promoter after blue light stimulation. Bulk assayed worms (multiple) were compared to individual assayed worms (single). *n = 15*, two-tailed unpaired Student’s *t* test: n.s. Blue bars represent “light ON.” Acetylcholine: *zxIs6 (Punc-17::ChR2::YFP),* GABA: *zxIs3 (Punc-47::ChR2::YFP).* *: *p* ≤ 0.05, **: *p* ≤ 0.01, ***: *p* ≤ 0.001

In liquid, photo-stimulation of ChR2 in GABAergic neurons inhibits swimming behavior, quantified as the decline in thrashing frequency [33]. The exact effects of cholinergic stimulation on swimming behavior, however, still need to be determined. Both swimming behavior and coiling behavior in liquid can be assessed with the WF-NTP [83], allowing other readouts, in addition to changes in body length, to be collected. Therefore, we analyzed thrashing frequency and eccentricity of worms in liquid expressing ChR2 under the *unc-17* or *unc-47* promotor to evaluate population-based optogenetic experiments with the OptoArm and the WF-NTP (Fig. 8A). We first explored the thrashing ability of worms grown with and without ATR before and during photo-stimulation. Photoactivation of ChR2 causes a decrease in thrashing behavior independent of which neuron population was stimulated (Fig. 8B). The kinetics of the observed movement decline was however strikingly different (Fig. 8C). GABAergic stimulation causes an initial decrease in thrashing behavior that gradually fades back to normal levels, while the cholinergic effect appears to saturate at the end illumination period (Fig. 8C). Indeed, it has been shown before that worms recover partially from paralysis even though illumination of GABAergic ChR2 is sustained [33]. Taking this difference in kinetics into account, we only analyzed the first 10 s of the movies for the worms with GABAergic ChR2-expression and the last 10 s for worms with cholinergic ChR2 expression (Fig. 8D). While the decline in thrashing behavior was already evident and significant when complete movies were analyzed (Fig. 8B), the effect was more pronounced when taking the underlying kinetics into account (Fig. 8D). Clearly, the population effect for GABAergic stimulation is most prominent directly after photo-stimulation while the cholinergic system requires more time for saturation of the effect. Importantly, at a blue light intensity of 1 mW/mm^2^, photoactivation of ChR2 in cholinergic neurons does not result in complete paralysis of worms in liquid (Fig. 8B–D). However, we did observe clear decreases in eccentricity when cholinergic neurons were stimulated via ChR2-based illumination (Fig. 8E), while the eccentricity was unchanged after GABAergic stimulation or when worms were grown without ATR (Fig. 8E,F). Again, we did observe that this effect could be maximized by long-term stimulation and analyzing the last 10-s of the assay. Interestingly, coiling behavior upon cholinergic stimulation has been ascribed to the concomitant GABA release that is indirectly triggered by photoactivation of cholinergic neurons innervating GABAergic neurons [33, 103–105]. Coiling behavior is completely absent when GABAergic mutants expressing ChR2 in cholinergic neurons are illuminated [33]. Therefore, coiling behavior likely provides a readout of the interplay between GABAergic and cholinergic neurons within the nervous system.

Finally, we investigated the use of multi-worm illumination and low-resolution (e.g., perimeters of 60–80 pixels instead of 2000–3000 pixels) videos to calculate the body length of illuminated worms expressing ChR2 cholinergically. While the variation between individual worms was much larger when multiple worms were illuminated at the same time, the average length of photoactivated worms was (0.92 ±0.08) strikingly similar to single-worm illumination (0.92 ± 0.02) (Fig. 8G). The variation is most likely caused by the lack of flat-field illumination of the OptoArm and, thus, while being small, generates spatial differences in light intensity (Figs. 3 and 4). Taken together, combining the OptoArm with a multi-worm tracker provides a way to perform population-based optogenetic experiments with multiple readouts in addition to body length. The coiling behavior and thrashing capacity are examples of such readouts and provide distinct information about the functionality of the neuromuscular system. However, it should be underlined that many other behaviors and associated readouts can be assessed when ChR2 is expressed in different neuronal populations, e.g., crawling speed for food slowing ([106]; possible with the WF-NTP [83]), reversals for touch-neuron stimulation, and egg-laying behavior after HSN-stimulation [57].

### A comprehensive optogenetic analysis reveals a faster age-dependent deterioration in the cholinergic system compared to other components of a neuromuscular unit

The ability to explore neuronal function with optogenetic tools provides an elegant way to study synaptic connectivity and function. As animals age, they exhibit a gradual loss in motor activity [107–111]. Over the years, the hunt for the mechanisms underlying this age-associated decline in movement has been subject to many studies [107–113]. For a long time, it has been hypothesized that the age-dependent decline in motor activity is the result of muscle frailty rather than a functional deficit in the nervous system (Herndon et al., 2002). This hypothesis was strengthened by studies showing that the structural integrity of the nervous system was found to be well preserved over time [110, 111, 114–117]. When other studies described mild morphological deteriorations at synapses at a later age, it was argued that the nervous system also undergoes are-related changed at the morphological level ([118–120]. In 2013, Liu et al. [113] questioned whether morphological analysis would be a reliable predictor for the functional status of a tissue. They showed that despite the preservation of integrity, motor neurons undergo an age-related functional decline in early life. At the same time, such a functional decline was absent in body wall muscles. Thus, they showed that neuronal dysfunction precedes muscle dysfunction [113].

**Fig. 9:**
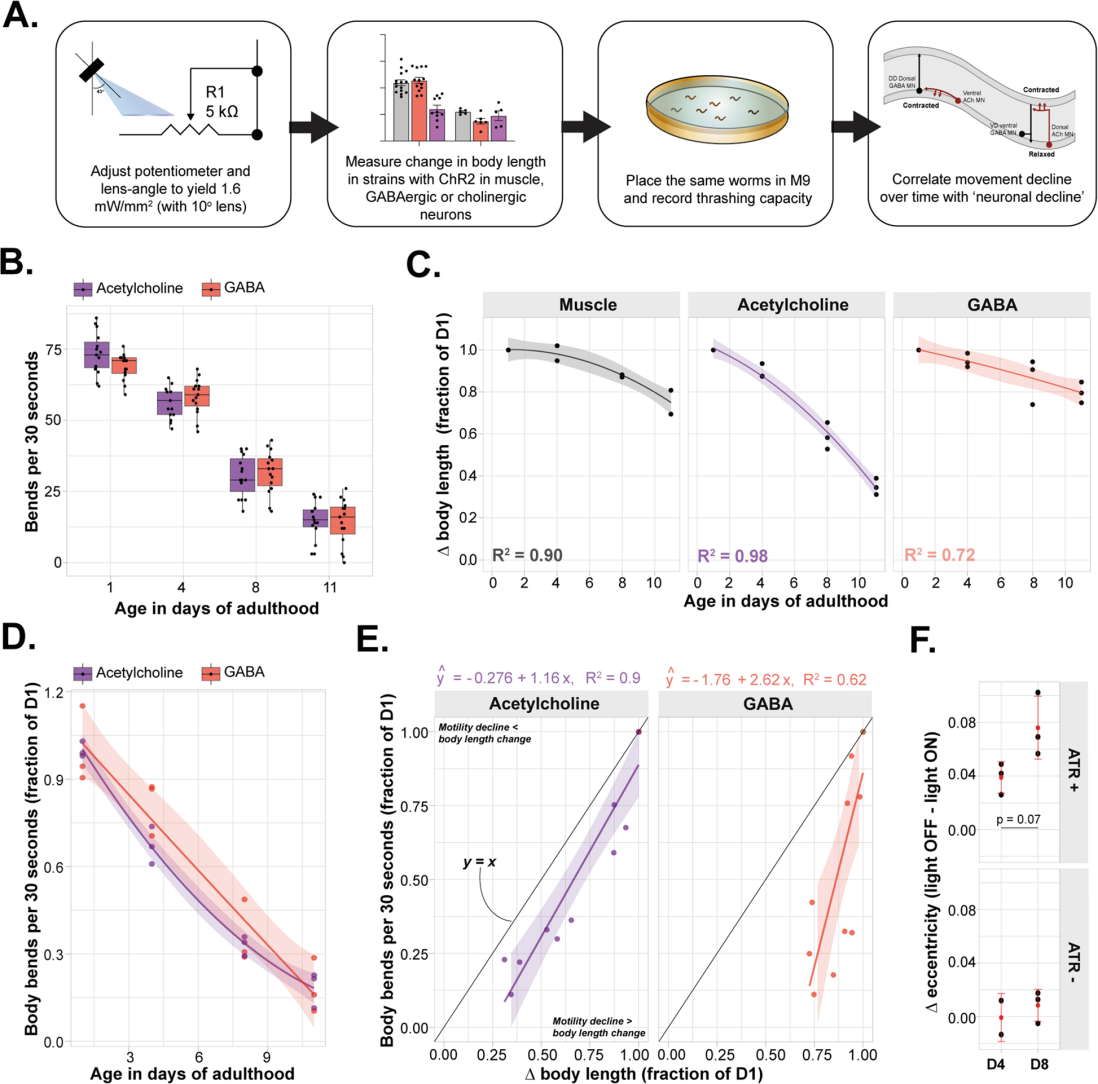
Cholinergic neuronal ageing correlates best with the decline in motility. **A** Schematic showing the experimental outline. The ∆body length was determined for strains expressing ChR2 in muscle, cholinergic neurons and GABAergic neurons at several time intervals. At the same time, the movement capacity, represented as bends per 30 s in liquid, was also measured. **B** The decline in thrashing capacity in liquid of worms expressing ChR2 in cholinergic or GABAergic neurons. *n = 15*, two-way ANOVA (Age: *p* < 0.001, interaction and neuron type: n.s.). The experiment was triplicated, one representative experiment is shown (see **D** for the averages of the other two experiments). **C** The normalized ∆body length (elongation for GABA, contraction for muscle and cholinergic neurons) at different ages as measured by the midline approach. Every point represents the mean of an experiment with > 10 biological replicates each. Fitted lines represent a general linear model with 2nd-order polynomial fit. All points within one experiment were normalized by the intra-experimental average at D1 within each strain. **D** The average decline in thrashing ability over time. Every point represents the mean of an experiment with > 15 biological replicates each. All points were normalized by the inter-experimental average at D1 within each strain. This sets the average bends per 30 s to exactly 1.0 at D1. Fitted lines represent a general linear model with 2nd-order polynomial fit. **E** The correlation between the ∆body length and ∆movement capacity (normalized to the changes and capacity at D1 intra-experimentally). Fitted lines represent simple linear regression without restrictions (both slopes deviate significantly from zero: *p* < 0.001). The black line corresponds to a relation of *x = y,* in which the decline in movement is equal to the decline in ∆body length. **F** The ∆eccentricity (eccentricity before illumination minus eccentricity during illumination) at different ages after optogenetic stimulation. Each individual point represents the mean of *n > 15* worms. Student’s *t* test: n.s. All experiments were replicated three times, all replicates are shown. The transparent zones around the fitted lines represent the confidence interval (95%). *: *p* ≤ 0.05, **: *p* ≤ 0.01, ***: *p* ≤ 0.001

While Liu et al. [113] shed light on the dynamics between the neuronal and muscle system during ageing, they did not specifically focus on the different types of motor neurons. As mentioned earlier, movement in *C. elegans* is the outcome of an antagonistic interplay between GABAergic inhibition and cholinergic stimulation [103, 104]. The lack of GABAergic input has severe consequence for the movement capacity [121, 122], while increased GABAergic stimulation has also been shown to results in cessation of thrashing behavior ([33]; Fig. 8). Clearly, the delicate balance between excitation and inhibition determines the capacity to move. While electrophysiology is technically challenging, optogenetics provide a relatively easy way of follow specific neuron populations functionally over time and relate those readouts with behavioral changes. Here, we investigated the age-related decline of both GABAergic neurons and cholinergic neurons specifically and correlated this decline with the deterioration of movement (Fig. 9A).

We started with assessing the movement decline over time by looking at the thrashing frequency of worms expressing ChR2 under the *unc-17* or *unc-47* promotor. As expected, we observed a significant age-dependent decline in thrashing frequency that is independent of the genotype (Fig. 9B). In order to study the relation between neuronal function and thrashing frequency, we assessed the “∆body length” of worms expressing ChR2 under the *unc-17* or *unc-47* promotor (e.g., elongation and contraction for GABAergic and cholinergic stimulation respectively) as readout for neuronal function after optogenetic stimulation with the OptoArm (1.6 mW/mm^2^) (Fig. 9C). Subsequently, the same worms were analyzed with the WF-NTP to assess their thrashing frequency (Fig. 9D). Different worms from the same population were tested at D1, D4, D8, and D11 of adulthood. Worms expressing ChR2 under the *myo-3* promotor were included as a control for “∆body length.” As previously described, we found that the postsynaptic muscle cells were relatively resistant to a functional decline in early life (Fig. 9C, Liu et al., 2003). The cholinergic neurons, however, revealed an almost logistic decline in function. Strikingly, this decline was not observed in GABAergic neurons (Fig. 9C). Then, we related the decline in thrashing frequency (Fig. 9D) with the decline in neuronal functioning (Fig. 9C) for each genotype (Fig. 9E). While the functional decline of neurons in neither of the motor neuron populations correlated perfectly with the decline in thrashing frequency, the cholinergic neurons had a slope of almost 1, suggesting a strong relationship between the decline in locomotion and neuronal function (Fig. 9E). Notice that the curve is slightly right-shifted (y = ax +b, b = − 0.267), which implies that, even though the *x, y* relationship is almost 1.0 (*x ~ y*), a portion of the decline in motility cannot be explained by cholinergic deterioration alone. Therefore, our data suggests that cholinergic-dysfunction correlates best with the age-dependent movement deficits. At the same time, we show evidence for different ageing kinetics between the cholinergic and GABAergic motor neurons. GABAergic neurons appear more resilient to ageing, suggesting that not all *C. elegans* motor neurons age equally fast.

Since a delicate balance between GABAergic and cholinergic function is essential for movement, the disbalanced decay between the two-neuron population might contribute to a changed excitation-to-inhibition ratio to the muscle. As stated before, due to the wiring of the nervous system, stimulation of cholinergic neurons normally causes a concurrent activation of GABAergic neurons that results in a typical coiling behavior [33, 103–105]; Fig. 8) and provides a readout that captures both systems at once. Therefore, we hypothesized that a changed balance between the GABAergic and cholinergic system can translate into a changed light-induced coiling propensity. In order to tackle this question, we assessed the change in eccentricity (as a readout for coiling propensity) induced by illumination of worms expressing ChR2 in their cholinergic neurons at D4 and D8, just before and after the steep decline of the cholinergic output (Fig. 9C). Although not significant, we observed a clear and consistent increase in “∆eccentricity” at D8 compared to D4 (Fig. 9F). The decrease in eccentricity is almost twice as high (Cohen’s *d*: 1.99) at D8. In conclusion, we hypothesize that the observed increase in coiling propensity could be due to an inequal decline of cholinergic and GABAergic neurons and thus might provide a subtle hint for a changed excitation-to-inhibition ratio towards inhibition.

### Automation and additional features can further increase the ease of use and functionality of the OptoArm

**Fig. 10:**
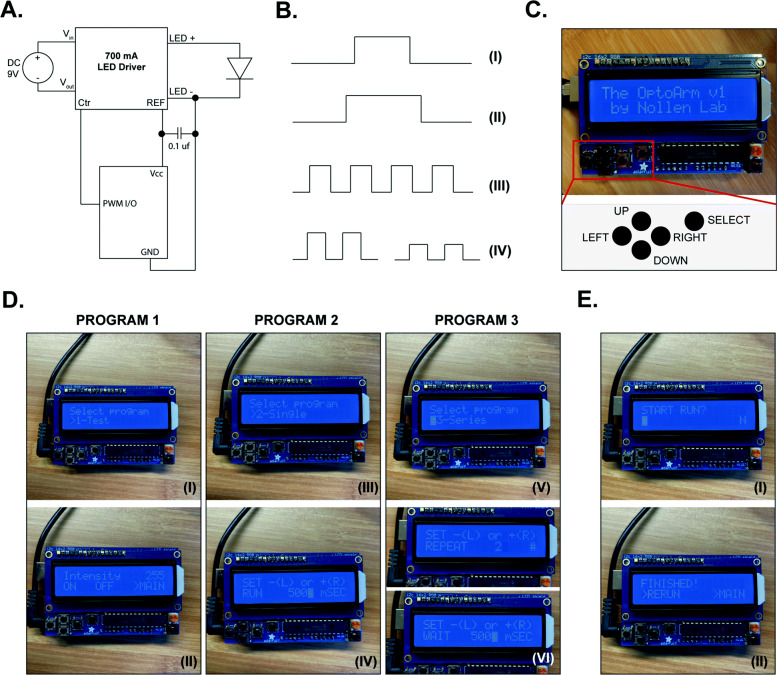
Automation of the OptoArm allows fine adjustment of light pulse width and intensity. **A** The electronic circuitry of the OptoArm under the control of a microcontroller (an Arduino Uno in this paper). **B** By using a pulse width modulator output pin of Arduino the pulse width (I, II), time intervals (III), and intensity (IV) of the OptoArm can be easily regulated. **C** By using an LCD shield on top of an Arduino Uno, all parameters can be adjusted in live-modus without the intervention of a computer. There are 5 buttons that can be used to go UP and DOWN in the different programs to select parameters and to adjust them by pressing LEFT or RIGHT. All changes can be confirmed with the SELECT button (see Table 5). **D** The different programs of the automated OptoArm. Program 1 (I) allows testing the system and manually turning the LED ON and OFF (II). Here, the intensity of the system can be changed as well. Program 2 (III) can be used to give single-timed pulses of light (IV). Program 3 (V) can be used to give trains of single-time pulses. One can adjust both the pulse-time (IV), the waiting time, and the number of cycles (VI). **E** The software provides a clear overview of the steps that are performed. When a run is started and then finishes, the user can use the option “Rerun” to execute the same program without having to adjust the parameters again

All optogenetic data collected in this paper was created with rather long photo-stimulation of at least 3 s. Those time intervals can be relatively well established by manual operation of the OptoArm, e.g., using the ON-OFF switch. The manual OptoArm can definitely be valuable for acquiring consistent optogenetic data as is evidenced by all data that is described in this paper (Figs. 7, 8 and 9), but its consistency is highly operator-dependent. Moreover, it is practically impossible to generate the short pulses or even high-frequency trains required for certain experiments through manual operation [33, 74]. Therefore, we explored the use of a microcontroller to circumvent the use of a computer and to provide a low-cost solution to the aforementioned problems. The 700 mA externally dimmable BuckPuck DC driver that is used to power the LED has a built-in 5 V reference/output to directly power the logic circuitry of a *u*processor without the need for an additional power source (Fig. 10A, Table 2). We used an Arduino Uno as a microcontroller to control the output of the OptoArm, making it a standalone system. By connecting a pulse width control pin (PWC, pin 9) to the CTRL pin of the driver, the Arduino can control not only the duration of a light pulse and the number of cycles, but can also adjust the light intensity, replacing the potentiometer (Fig. 10B). These three parameters are easily adjustable by reprogramming of the Arduino software. However, adjusting Arduino scripts requires both knowledge of the programming language and a computer. Since we have tried to keep the OptoArm a standalone set-up that can be easily operated without specialistic knowledge and expensive equipment, we propose a more elegant solution. We decided to use an LCD shield on top of the Arduino to adjust different parameters in live-modus without the intervention of a computer (Fig. 10C). Then, we created an Arduino script that allows users to use the LCD screen and connected buttons to select different options and menus (Supplementary software 1, Tables 5 and 6). The software provides 3 programs through which the users can navigate for different experimental purposes: a testing program, a single run menu, and a series pulse menu (Fig. 10D,E, Table 6). When using the automated OptoArm, one has to build the circuitry depicted in Fig. 10A and upload the provided software to the Arduino only once (connect the CTRL pin of the LED driver to pin 9 of the Arduino to ensure compatibility between the software and the circuit). The used LCD shield is relatively expensive when compared to the other components of the system, but it offers a user-friendly solution (Tables 5 and 6). Nevertheless, other LCD screens can be used without a problem, as long as the software is adjusted as well.

**Table 5:** Arduino controls and button functions

Display screen	Controls	Function	Fig. 10
**Main**	UP/DOWN	Scroll through program options	D: I,III,V
	SELECT	Confirm selection	
**Program 1 settings**	UP/DOWN	Set intensity	D: II
	LEFT/RIGHT	Switch between ON/OFF/MAIN	
	SELECT	Confirm return to main menu	
**Program 2/3 settings**	LEFT/RIGHT	Set parameter value	D: IV, VI
	SELECT	Confirm settings, skip to next display	
**Program 3 settings**	UP/DOWN	Switch between parameters	D: VI
**Start run**	LEFT/RIGHT	Switch between yes/no	E: I
	SELECT	Confirm selection	
**Finish**	LEFT/RIGHT	Switch between rerun/return to main	E: II
	SELECT	Confirm selection

**Table 6:** The different programs within the OptoArm software

*Program 1:* The testing menu. This menu is used to set-up your experiment. The menu allows the user to select the intensity of the LED by changing the analog scale: 0–255 (buttons up and down) and to turn the LED ON at that intensity. This menu is ideal for adjusting the working distance and incidence angle to gain the appropriate intensity before starting an actual experiment. Importantly, as long as the Arduino is powered, the set intensity will be saved and the standard for the other menu options (Fig. 10D—I/II, Table 3). *Program 2:* The single run menu. In this menu, the user can select the time of the light pulse (Fig. 10D—III/IV). The buttons are time-sensitive: the longer one pushes, the steeper the steps. There is standard a 1-s waiting step before the pulse when the run is started. When the run is completed, the users get the opportunity to rerun the assay, without setting the parameters again (Fig. 10E, Table 3). *Program 3:* The series pulse menu. In this menu, the user light pulse duration, the waiting time (between two light pulses), and the number of cycle repeats (Fig. 10D—IV-VI). By going up and down in the menu, the different parameters can be adjusted (Table 3). There is again a 1-s waiting step before the first pulse when the run is started. When the run is completed, the users get the opportunity to rerun the assay, without setting the parameters again (Fig. 10E).	

It has been underlined now several times that the OptoArm provides the user with an adaptable system for optogenetic experiments. The ease of automating the OptoArm strengthens that statement, but there is more to add. First of all, the system uses a Royal-Blue LED because ChR2 requires a wavelength in the blue spectrum. There are however many more, inhibitory light-gated ion pumps or outward-directed proton pumps that can be used for optogenetic purposes [37, 60, 123–125]. The yellow-green light-sensitive archaerhodopsin-3 (Arch) [123], halorhodopsin from Natronomonas pharaonic (NpHR) [37, 60], and rhodopsin from Leptosphaeria maculans (Mac) [124, 125] are a few examples of the light-sensitive actuators that are widely used. NpHR, Arch, and Mac are activated by wavelengths of 580–600 nm, 568 mm, and 540 nm respectively [60]. By substituting the royal blue (448 nm) LED with a lime variant (567 nm), the OptoArm can also be used to photoactivate those inhibitory rhodopsins (Table 2). If one would be interested in switching between colors, we highly recommend to either build two arms, or to wire the OptoArm in such a way that only the heatsink and the attached LED can be disconnected and exchanged. Finally, while the OptoArm was specifically developed to work with *C. elegans*, it can also be used for experiments with *Drosophila melanogaster*. At present, required intensity for optogenetic stimulation of *D. melanogaster* is nowadays a few orders of magnitudes smaller. Commonly used intensities range from 50 *u*W/mm^2^ to 0.3 mW/mm^2^, although much higher intensities (< 6.5 mW/mm^2^) are also used [126–128]. To gain such small intensities with the OptoArm, one can use the systems intensity control, omit the use of lenses, and/or increase the working distance. In addition, with such a high sensitivity to light, the effect of the backlight of recording systems (e.g., microscope and tracker lights) should be investigated as well.

## Discussion

While pharmacological tools and electrophysiology provide an unprecedented way to analyze synaptic function in *C. elegans*, optogenetic tools have revolutionized the way we can study neuronal circuits in a more noninvasive way [31]. The expression of light-sensitive ion channels and pumps under tissue or cell-specific promotors, allow researchers to remotely modify and manipulate the behavior of excitable cells [31, 32, 37–43]. Consequently, the ability to tweak synaptic activity and concurrently observe its effect on behavioral readouts makes it possible to dissect the neuronal circuits underlying animal behavior. While many systems are available for optogenetic purposes [57, 58, 62–64, 66–68, 70–74], the associated costs, the required manual adaptations, compromises for usability, and low adaptability provide a major bottleneck in the selection of the system. Here, we argue that optogenetics should be made accessible to all researchers regardless of limited financial resources or advanced technical expertise. Therefore, we designed a low-cost optogenetics system that provides high-quality and consistent experiments, while also being easy and flexible in useability.

We have developed the OptoArm as an inexpensive and simple to set-up system for the optogenetic stimulation of *C. elegans*. It provides the user with adjustable light intensity and lightning profiles via the incorporation of interchangeable lenses. The ability to change lenses allows optogenetic manipulation and analysis of the resulting behavior in both single worms and entire worm populations. Therefore, we technically validated the system in different set-ups, ensuring compatibility with both standard microscopes and existing worm trackers like the WF-NTP. The compactness of the system makes it easy to incorporate in a variety of experimental set-ups, as the only requirement is a power outlet and minimal space. In addition, the OptoArm can be easily automated by the inclusion of a microcontroller, thereby allowing users to perform more complex optogenetic experiments involving high-frequency trains of light pulses. By offering both the option to build a manual and a fully automated system, researchers are able to pick the set-up that fits their budget and requirements best. The cheapest version of the OptoArm only costs $65 dollar, while the variant with an included stand will take about $ 90 dollars in total. The fully automated system, including stand, is set to only ~ $128 dollar, thereby outperforming most other optogenetic devices available. In addition, the capabilities of the system can be expanded by low-cost additional modules as the need presents itself, making it an investment for the long term as well.

Considering the fact that overall costs are often a key determinant in adopting a new device, the OptoArm circumvents a major impediment. Next to its flexibility and adaptability, the OptoArm also bypasses the need of extra adjustments that are often required for low-cost systems, like aluminum foil or closed boxes, which are often incompatible with live imaging systems. Therefore, the OptoArm provides a way to illuminate and record worms simultaneously. Nevertheless, there are also some disadvantages when comparing the OptoArm with more expensive optogenetic systems [57, 58, 62–64, 66–68, 84]. First of all, the OptoArm does not allow to follow single worms in space with both temporal and chromatic precision. It does not allow illumination at a specified anatomical position only. Therefore, it is not possible to excite single neurons with the OptoArm. Nonetheless, this is only required for very specific, specialist applications. Secondly, our current configuration of the OptoArm provides an adaptable light intensity up till ~1.8 mW/mm^2^ (with a fixed working distance of 3.5 cm), but some experimental procedures require a higher intensity up till 5 mW/mm^2^ [31]. However, it is possible to extend the system with a tri-LED that triples the power of the available light. In this way, with the appropriate thermal management and lenses, the light intensity of the OptoArm could be further increased. Thirdly, while it is possible to perform multi-worm illumination with the OptoArm, the spatial consistency is not ideal. For plates requiring illumination of a small surface area (e.g., 3 cm, 12 wells), the OptoArm can provide a coverage of sufficiently consistent light intensity. Yet, when larger areas are illuminated, the light gradient and intensity drop towards the periphery becomes more of an issue. Lastly, the OptoArm cannot shift between wavelengths, as would be possible with a fluorescence microscope. Nevertheless, it is possible to change illumination color by incorporating different types of LEDs. In this way, the OptoArm could not only be used for photoactivation of ChR2, but also for Mac, NpHR, or Arch [60]. Clearly, the modular nature of the OptoArm can be used to overcome some of the mentioned limitations.

While some disadvantages exist, we still demonstrated that the OptoArm can accurately reproduce previously established findings that were acquired with more expensive optogenetic systems. Furthermore, by using the OptoArm with the WF-NTP a variety of population characteristics can be acquired on both solid and liquid media, despite some spatial inconsistency in illumination. We also show that the OptoArm can be used to tackle novel questions by combining different optogenetic readouts. In fact, we used the OptoArm to study the age-related decline of specific motor neurons and the accompanying deterioration in locomotion. We found a striking correlation between the age-dependent decline in cholinergic output and the decline in thrashing capacity. However, the decrease in cholinergic output, as evidenced by a change in body length upon optogenetic stimulation, did not completely explain the drop in movement capacity during ageing, neither did the decrease in GABAergic output or muscle function. In fact, we observed that the muscular and GABAergic system appeared to be more resilient to age-related deterioration. Indeed, the age-related decrease in GABAergic output was much smaller than that of the cholinergic branch. It has been previously shown that the overall function of a neuromuscular unit decreases early in life due to neuronal deficits rather than of muscle dysfunction [113]. However, to our knowledge, the striking differences in ageing kinetics between GABAergic and cholinergic neurons have not been described before.

The GABAergic and cholinergic system are inextricably linked and regulate the fine balance of excitation-to-inhibition stimuli to the muscle, ensuring coordinated movement [103, 104]. If GABAergic neurons are indeed more resilient to ageing, while cholinergic neurons gradually lose their functional capacity, the balance of the excitation-to-inhibition ratio for muscle cells might also change. Intriguingly, given the underlying interaction between GABAergic and cholinergic neurons that shapes the coiling phenotype [33], the observed light-induced increase in coiling behavior at later ages might actually provide the first hint for an altered excitation-to-inhibition ratio. While the age-dependent decline in locomotion correlates well with a decrease in cholinergic signaling, it might be enhanced by the much slower decline in GABAergic function. To add another layer of complexity, a study by Liu et al. [113] found evidence of postsynaptic sensitization, increasing the amplitude of both acetylcholine- and GABA-evoked muscle currents during early ageing and peaking at D9. The relationship between these postsynaptic adaptation mechanisms and presynaptic changes will further determine the delicate balance of the neuromuscular unit and the overall movement capacity. Clearly, further research to unravel the complex interplay that contributes to age-related decrease in movement capacity is required and should integrate all these components instead of focusing on a single compartment. Moreover, comparing GABAergic and cholinergic neurons molecularly might provide insight into the key players underlying the differences in resilience to ageing.

## Conclusion

Here, we have presented a low-cost, easy-to-build, and highly adaptable optogenetic device that allows researcher to perform novel optogenetic experiments with *C. elegans* and other small organisms. We validated the OptoArm technically and biologically in different set-ups for both single and multiple worm experiments. The flexible and adaptable nature ensures compatibility with different recording systems, allowing different readouts to be combined for more elaborate insight in biological systems. The OptoArm can be easily operated without expert knowledge and, due to its modular nature and cheap components, can be easily adapted as well. In the end, users have ultimate control over their budget and system configuration. Altogether, we show that the OptoArm is able to overcome some of the major obstacles preventing a more widespread implementation of optogenetics, including in teaching institutes.

## Methods

### Strains and maintenance

Standard conditions were used for *C. elegans* propagation at 20 °C [129]. Animals were synchronized by hypochlorite bleaching, hatched overnight in M9 buffer, and subsequently cultured on NGM agar plates seeded with OP50. For optogenetic experiments, transgenic worms were cultivated in the dark at 20 °C on NGM plates with or without 0.2 mM all-trans retinal (ATR) added to the OP50. A final concentration of 0.2 mM ATR was obtained by mixing 0.4 μl of a 100 mM ATR stock solution in ethanol (Sigma-Aldrich) with the 200 μL *E. coli* that was spread on each 6-cm plate. For ageing experiments, worms were transferred ~ 72 h after plating to NGM plates containing 5-fluoro-2′deoxy-uridine (FUdR) to inhibit growth of offspring. For ageing experiments until adulthood D12, worms were transferred every 3 days to fresh ATR+ and ATR− plates. The following genotypes were used: ZX388: zxIs3[*unc-47p::ChR2(H134R)::YFP + lin-15(+)]*V, ZX460: zxIs6[*unc-17p::ChR2(H134R)::YFP + lin-15(+)*]V, ZX463: *unc-47(e307)*III; *zxIs3*, ZX465: *unc-26(s1710)*IV; *zxIs3*, ZX511: *unc-26(s1710)*IV; *zxIs6*, ZX531: *unc-47(e307)*III; *zxIs6*. All strains were a kind gift of professor A. Gottschalk.

### The OptoArm

#### Required components

All the components of the Optoarm are by Quadica Developments, unless stated differently. For the electronic circuitry, we refer to Fig. 2A. The OptoArm consists of a Royal-Blue (448 nm) Rebel LED on a SinkPAD-II 20 mm Star Base of 1.03 W (SP-01-V4), powered by a 700 mA externally dimmable BuckPuck DC driver—with leads (3023-D-E-700), and connected to a connecting wire with adjustable potentiometer of 5 kΩ (3021HEP). The LED is attached to a heatsink (50 mm round × 44 mm high alpha heatsink—5.2 °C/W; CN50-40B) with pre-cut thermal adhesive tape for 20 mm Star LED assemblies (LXT-S-12). The lens holder was self-manufactured from aluminum with a Schaublin 125 lathe (*ϕ* 21 mm, inner circle) and contains a small screw hole at the side (M2) for tightening and loosening lenses and two grooves for the LED wires (Fig. 2B). The holder was attached to the heatsink with mounting tape for Khatod 20 mm Round optic Holders (LT-06). Three different lenses (with adjacent integrated holder) were used: Khatod 10° 22 mm circular beam optic (KEPL115406 by Khatod Optoelectronic), Fraen 21° 22 mm circular beam optic (FLP-M4-RE-HRF, by Fraen Corporation), Khatod 40° 22 mm circular beam optic (KEPL115440 by Khatod Optoelectronic). For the ON/OFF switch, a latching pressure switch (≤ 3A, ≤ 250 V; Velleman – R1821A-RD) was used. The whole system was mounted on a three-pronged clamp (*ϕ* 10 mm, 241-7432, Usbeck Carl Fried). A cross-clamp (105 BR 10, Comar) was placed on the rod of the clamp. The clamp provides a way to attach a standard DC contra plug (≤ 4A, ≤ 25 V, Velleman; CD021) and to power the system with a DC adapter. Due to the nature of a LED driver, the input voltage to the driver must always be higher than the total forward voltage drop of all connected and consuming components. In this case, the driver has a minimal input margin of 2.5 V and the LED consumes about 3.5 V, leading to a minimal input voltage of 6 V. However, when working with a potentiometer, it is highly recommended to use a minimum of 7V_DC_ (as evidenced by the spec sheet). We have been using 9V_DC_, 12V_DC_, and 15V_DC_ adapters (~ 1.0–1.2 A) with good results. All components can also be found in Table 2. Clearly, one can substitute the potentiometer, switch, and DC connector for cheaper variants. The critical parts of the system include the LED, heatsink, and steady 700 mA driver together with the lenses.

#### Automated OptoArm

For the automated OptoArm, the aforementioned components are extended with an Arduino Uno (or any clone), a capacitor of 0.1 μf (Xicon Ceramic Disc Capacitors; Mouser electronics, 140-50 U5-204M-RC) and an LCD Shield kit w/ 16 × 2 character display (Adafruit, ID:714), while the connecting wire with adjustable potentiometer of 5 kΩ can be omitted. The shield has to be assembled with some soldering steps; a great tutorial can be found here: https://learn.adafruit.com/rgb-lcd-shield/assembly. See Fig. 10A for the electronic circuitry. The accompanying Arduino code for the LCD shield is freely available and can be downloaded from: Supplementary Software 1 (the software runs under the license of Attribution-NonCommercial-ShareAlike 4.0 International (CC BY-NC-SA 4.0). When using this software, connect pin D9 of the Arduino to CTRL pin of the LED driver.

#### Stands

For experiments with the WF-NTP, we used a stand that was built with the following components: stand rod (1000 mm, *ϕ* 12 mm, 241-7153, Usbeck Carl Fried), double bosshead (241-7104, Usbeck Carl Fried), retort stand base, tripod (241-0245, Usbeck Carl Fried). For experiments with a microscope, we used a stand with the following components: basic carrier (20 RM 01, Comar), Pinion stage (30 XT 40), post-holder (45 BH 10), rod (203 RM 01), self-manufactured base.

### Microscope and WF-NTP set-up

#### Microscope

A high-resolution microscopy system was used to image the worms, consisting of an Olympus SZ51 microscope coupled with an IphoneX via a Carson HookUpz 2.0. smartphone adapter. The UV protection shield of a Leica MZ 16 FA was used to avoid saturation of the camera. In general, a 1.7× digital zoom (of the Iphone) was used in combination with a × 15–30 magnification of the microscope, and videos were always acquired with a framerate of 30 fps. Actual working distances were determined by measuring the height from the surface perpendicular to the middle of the LED and using the trigonometric ratio for sine, eq. 8, to calculate the length of the hypotenuse side:

8 $$ \sin \left(\theta \right)=\frac{\mathrm{opposite}}{\mathrm{hypotenuse}} $$

A working distance of 3.5 cm is often adequate.

#### WF-NTP

The used set-up was identical to the one described in Koopman et al. [83], using a camera-to-sample distance of 140 mm. The used framerate was always 20 fps and the working distance was set to ~ 4.1 cm. In both set-ups, light intensity was always measured at 448 nm with a M16-130 USB power meter (Thorlabs, *ϕ* 9.5 mm) to verify the required conditions. Before each experiment, we measured the light intensity to ensure appropriate conditions, assuming a Gaussian light source (in the software).

### Behavioral experiments

For single-worm illumination, worms were freely moving on unseeded 3-cm NGM plates and kept in frame by manual location. For “short-term” experiments, worms were exposed to a 3-3-3 lightening regime, in which light was OFF for 3 s, followed by light ON for 3 s and light OFF for another 3 s. Light intensity was always 1.6 mW/mm^2^ (10° lens) unless stated differently. For “long-term” experiments, worms were exposed to a 1-50-1 lightening regime in which light was OFF for 1 s, followed by light ON for 50 s and light OFF for another second. For multiple-worm illumination, worms were collected in M9 buffer and plated on an empty 3-cm plate that was flooded with 1.5 mL M9. Worms were recorded with the WF-NTP set-up at a framerate of 20 fps and a light intensity of 1.0 mW/mm^2^ (21° lens) unless stated differently. We generated separate movies for light OFF (30 s) and light ON (30 s). For most experiments, as for the ageing-timeline, worms were first examined via single-worm illumination and subsequently transferred to M9 for multi-worm illumination.

### Image processing

Videos of multiple-worm illumination, acquired with the WF-NTP platform, were analyzed with accompanying software as described in Koopman et al. [83]. Videos that were acquired with a cellphone (Iphone X) were transferred to a computer (.mov) and first converted to Fiji compatible files (.avi) with ffmpeg. We used a terminal (cmd for Windows, terminal for MacOS) to navigate to the directory with the movies and quickly convert multiple movies. When opening a terminal, the current location will be visible (e.g., C:\Users\). When the movies are saved at a different disk, for example, D, one can use the command: C:\Users\Tracker>**d**: followed by pressing “Enter” to switch directly to that disk. Paths can be changed with the command **cd\**, for example, C:\Users\Movies>**cd\** followed by pressing “Enter.” This will yield “C:\Users.” Adding paths is managed by typing cd **X**, for example, C:\>cd **Movies** followed by pressing “Enter.” This will yield “C:\**Movies**.” When the appropriate directory is selected, the following command can be executed:

$$ \mathrm{for}\ \mathrm{i}\ {\mathrm{i}\mathrm{n}}^{\ast }.\mathrm{MOV};\mathrm{do}\ \mathrm{ffmpeg}-\mathrm{i}"\$\mathrm{i}"-\mathrm{pix}\ \mathrm{fmt}\ \mathrm{n}\mathrm{v}12-\mathrm{f}\ \mathrm{avi}-\mathrm{vcodec}\ \mathrm{rawvideo}"\$\left\{\mathrm{i}\%.\ast \right\}.\mathrm{avi}";\mathrm{do}\mathrm{n}\mathrm{e} $$

This code converts all .MOV files in the directory to .AVI files at the same time. For the perimeter approach, the converted movies were processed with Fiji [96]. Movies were first set to an 8-bit format (Image > Type > 8-bit) and then duplicated (Image > Duplicate) to ensure accessible images for all subsequent steps. Next, the movie contrast was enhanced by selecting 0.3% saturated pixels and normalized enhancement (Process > Enhance), followed by background subtraction with a rolling ball radius of 50 px (Process > Subtract Background). Then, we thresholded the images (Image > Adjust > Threshold) by the Huang method (same threshold for the whole stack) and applied the settings to all frames. Finally, we measured the perimeter of the worm per frame by setting the measurements (Analyze > Set measurements) to perimeter only and clicking on “analyze particles” (Analyze > Analyze particles). One should set a lower limit of the size of the particles to avoid noise being picked up. This number highly depends on the used magnification and should therefore be determined by an empirical approach. We recommend to record the first processing steps until subtracting the background (Plugins > Macros > Record) and created a macro (Create) that can be rerun for every movie. In this way, only the thresholding had to be performed manually. Measurements were normalized by the average pixel length of the perimeter measured in the first 30–60 frames (light OFF). For long-term experiments, a midline approach was used at specified intervals (0.5 s, 2 s, 10 s, 20 s, 30 s, 40 s, 50 s, and 52 s). If the midline approach was used for short-term experiments, we measured the body length 500 ms before light was ON and 1000 ms after light was turned ON. When the midline approach is specifically mentioned, we measured the body length by drawing a midline from head to tail with a drawing-tablet (Wacom).

### Statistics and visualization

Statistical analyses were done in R and GraphPad Prism 8. The used statistical tests can be found in the different figures and are based on several criteria. In short, (log-)normality tests were always performed on the collected data to test for Gaussian distributions (e.g., Shapiro-Wilk). Based on the distribution of the data, the appropriate test was selected (parametric or non-parametric). When more than two groups were compared, multiple-testing correction was always applied. When inequality of variance was expected between two groups (based on the experimental design), Student’s *t* tests were always performed with a Welch’s correction (The Welch test must be specified as part of the experimental design, and not decided upon a posteriori [130, 131]. Post hoc testing after a one- or two-way ANOVA was only performed when the initial test gave significant results. All experiments were replicated three times, unless stated differently. *: *p* ≤ 0.05, **: *p* ≤ 0.01, ***: *p* ≤ 0.001. All data was visualized with R (ggplot2) or Graphpad Prism 8 and color-adjusted in Adobe Illustrator.

## Supplementary Information

**Additional file 1.**

## Data Availability

All data generated or analyzed during this study are included in this published article and its supplementary information files. The raw movies are available on request from the corresponding authors and from the DataverseNL repository at 10.34894/89DM6O.
