# Publisher Correction to: An economical and highly adaptable optogenetics system for individual and population-level manipulation of *Caenorhabditis elegans*

**DOI:** 10.1186/s12915-021-01152-8

**Published:** 2021-09-27

**Authors:** M. Koopman, L. Janssen, E. A. A. Nollen

**Affiliations:** European Research Institute for the Biology of Ageing, University of Groningen, University Medical Center Groningen, Groningen, The Netherlands

**Publisher Correction to: BMC Biol 19, 170 (2021)**

10.1186/s12915-021-01085-2

Following publication of the original article [1], the authors noted that incorrect Additional files were published, due to a typesetting mistake. The correct version of the single Additional file for this article (Additional file 1) is attached to this Publisher Correction and original article has been updated.

The publisher apologises to the authors and readers for the inconvenience caused by the error.

## Supplementary Information

**Additional file 1.**
